# Machine Learning Prediction Models for Preeclampsia: Systematic Review and Meta-Analysis

**DOI:** 10.2196/78714

**Published:** 2026-01-19

**Authors:** Lu Liu, Qixuan Zhu, Yichi Zong, Xueyuan Chen, Wei Zhang, Jun Wang

**Affiliations:** 1 School of Public Health China Medical University Shenyang City, Liaoning Province China; 2 Department of the Obstetrics and Gynecology Shengjing Hospital Affiliated to China Medical University Shenyang City, Liaoning Province China China; 3 School of Engineering University of Pennsylvania Philadelphia, PA United States; 4 Department of the Obstetrics and Gynecology Shengjing Hospital Affiliated to China Medical University Shenyang City, Liaoning Province China

**Keywords:** preeclampsia, machine learning, predictive models, meta-analysis, artificial intelligence, computer-assisted diagnosis

## Abstract

**Background:**

Preeclampsia is a severe hypertensive disorder with rising global prevalence. While machine learning (ML) models for predicting preeclampsia are increasingly published, existing evidence shows high heterogeneity, and the distinction between internal performance and external transferability remains unclear.

**Objective:**

This study aims to evaluate the performance of ML models in predicting preeclampsia through a systematic review and meta-analysis, while also exploring their potential clinical application value, in order to specifically enhance the quality of future research and the predictive capability of the models.

**Methods:**

Following PRISMA (Preferred Reporting Items for Systematic Reviews and Meta-Analyses) guidelines and PROSPERO registration, we searched PubMed, Web of Science, IEEE Xplore, and CNKI (China National Knowledge Infrastructure) for studies published through February 2025. We included studies using ML to predict preeclampsia in pregnant women. Bias was assessed using PROBAST (Prediction model Risk of Bias Assessment Tool). We calculated summary estimates using random-effects models and, crucially, computed 95% prediction intervals (PIs) to estimate performance in future clinical settings. Subgroup and meta-regression analyses were conducted to explore heterogeneity.

**Results:**

In total, 26 studies comprising 31 ML models were included. While the pooled area under the receiver operating characteristic curve was high at 0.91 (95% CI 0.87-0.92), extreme heterogeneity was observed (*I*^2^>99%). The 95% PI for sensitivity was wide (0.32-0.96), indicating that in some external settings, sensitivity could drop to 32%. Only 6 studies conducted external validation; in these, the pooled sensitivity decreased to 0.68, with a PI of 0.25-0.94.Subgroup analysis suggested that models incorporating laboratory biomarkers and neural networks outperformed others, though CIs overlapped.

**Conclusions:**

Current evidence suggests that a high area under the curve in ML models is more likely to reflect the “performance” of the model on the internal development dataset rather than its universal “effectiveness” and clinical utility in independent, diverse populations. The apparent performance exhibits significant contextual dependence. Future studies should conduct multicenter, prospective external validation and recalibration research to enhance transferability and reliability.

**Trial Registration:**

PROSPERO CRD420251005830;https://www.crd.york.ac.uk/PROSPERO/view/CRD420251005830

## Introduction

Preeclampsia is a pregnancy-related hypertensive condition marked by the development of high blood pressure and protein in the urine after 20 weeks of gestation. Due to its multiple etiologies and complex pathogenesis, it poses significant risks to both maternal and perinatal health [1]. This specific condition negatively impacts maternal health and can also lead to serious complications for the fetus, including placental abruption and restricted fetal growth. According to global statistics, the incidence of preeclampsia ranges from 3% to 9%, with even higher rates observed in certain high-risk populations [2]. Furthermore, preeclampsia is one of the leading causes of maternal mortality worldwide, particularly in low- and middle-income countries. The prevalence of preeclampsia in China has increased from 5.79% in 2005 to 9.5% in 2019 [3], further underscoring the urgent need for early screening and management. To date, the etiology and pathogenesis of preeclampsia remain incompletely understood, and effective treatment measures are lacking. Consequently, early detection and enhanced management are essential clinical strategies.

Understanding the epidemiological characteristics of preeclampsia is essential for developing effective public health strategies. In the study of preeclampsia, traditional statistical methods primarily emphasize linear models and hypothesis testing, which are effective in uncovering singular relationships between variables. However, the pathological mechanisms underlying preeclampsia are highly complex, involving multiple interacting factors, and traditional methods may face limitations when addressing nonlinear and high-dimensional data. In contrast, machine learning (ML) technology has shown considerable promise in this domain.

A subset of artificial intelligence (AI), ML is a technology that allows computers to independently learn from data and make decisions or predictions using algorithms and models. Its application in clinical settings can effectively prevent and manage diseases. Currently, the usage of ML to develop predictive models for preeclampsia is becoming increasingly prevalent. For instance, Sylvain et al [4] noted that the implementation of ML methods has significantly improved the prediction accuracy of high-risk pregnancies, offering a novel perspective for the early identification of preeclampsia. Furthermore, Ranjbar et al [5] indicated that ML-based models surpass traditional regression models in predicting the incidence of preeclampsia. The multidimensional optimization capabilities of these models allow them to account for interactions among various clinical features and biomarkers, thereby enhancing diagnostic accuracy.

By leveraging ML, researchers can explore both linear and nonlinear relationships, as well as uncover deep-seated features and patterns within the data. This method establishes a scientific foundation for the prompt recognition and intervention of preeclampsia.

Compared with prior systematic reviews and protocols on pregnancy outcomes or preeclampsia, the incremental contributions of this study are as follows: (1) we prespecified and implemented subgroup analyses by outcome definition, gestational window, data source, and validation type to avoid indiscriminate pooling across highly heterogeneous models and populations; (2) we treated area under the curve (AUC) as the primary summary measure and applied robust univariate random-effects models (Hartung-Knapp-Sidik-Jonkman method) to pool sensitivity and specificity separately, accompanied by 95% prediction intervals (PIs) to estimate future performance; and 3) we clearly separated performance in internal vs external validation and documented whether decision-curve analysis was conducted. Taken together, these methodological enhancements aim to provide more interpretable evidence about where deployment may be appropriate and where it remains premature.

## Methods

### Research Design

This research was carried out in alignment with the PRISMA (Preferred Reporting Items for Systematic Reviews and Meta-Analyses) 2020 standards [6] (Multimedia Appendix 1 [7]). Specific details regarding the search keywords can be found in Textbox S1 of the Multimedia Appendix 2. Before the study began, the protocol received approval and was registered with the PROSPERO under the reference number CRD420251005830.

### Literature Search Strategy

Comprehensive searches were executed in several prestigious databases, including PubMed, Web of Science, IEEE Xplore, and the CNKI (China National Knowledge Infrastructure). These searches focused on locating scholarly papers that were published in either English or Chinese. The time frame for this search encompassed works published until February 2025, ensuring that the most recent and relevant literature was included in the investigation. The search strategy was developed based on the PICO (Population, Intervention, Comparison, and Outcome) framework. In this study, “P” denotes the population with PE, “I” refers to ML methods as the intervention, “C” indicates the gold standard for comparison, and “O” encompasses outcomes, such as sensitivity, specificity, and accuracy for prediction and diagnosis (Table S1 in Multimedia Appendix 2). Additionally, the reference lists from each identified study underwent a manual review to uncover further relevant research. Zotero (Center for History and New Media at George Mason University) was used to organize the studies and remove any duplicates.

The study’s inclusion criteria were formulated to guarantee the rigor and relevance of the research. The criteria encompassed (1) research papers published in English or Chinese; (2) investigations involving pregnant women from the general population that explicitly defined the diagnosis of preeclampsia; (3) studies that used ML models for predicting preeclampsia, along with a thorough explanation of these models; and (4) investigations that showcased the performance of the ML models, offering adequate data to determine both sensitivity and specificity. These criteria aimed to strengthen the validity of the results and ensure a thorough assessment of the existing literature.

The exclusion criteria for this study are as follows: (1) studies that solely investigated risk factors without developing a predictive model; (2) papers published in languages other than English or of types other than original research, such as reports and reviews; (3) duplicate publications; (4) studies that included 2 or fewer predictors in the constructed model; and (5) studies for which the full text was not accessible.

### Literature Screening and Data Extraction

Five researchers (LL, QZ, YZ, XC, and WZ) meticulously followed the established inclusion and exclusion criteria to screen the titles and abstracts of the literature. Studies that met these criteria advanced to the full-text reading phase, where all relevant studies were reviewed. Each article underwent a minimum of 2 rounds of screening. Both the title and abstract screening, as well as the full-text reading, were conducted independently by the 2 researchers (LL and QZ). In instances of disagreement between them, another researcher (JW) made the final decision.

In total, 26 studies [8-33] were chosen for analysis. Data extraction was independently performed by 2 researchers (LL and QZ) following the standardized protocol established by the TRIPOD (Transparent Reporting of a Multivariable Prediction Model for Individual Prognosis or Diagnosis), as outlined in the existing literature [34]. Data collected from each study included the following: (1) demographic details, such as the country of data collection, the study setting, the source of the data, the design of the study, and the definition of outcomes; (2) methods for data partitioning, feature selection algorithms, types of ML prediction models, model validation, and applications; (3) results of predictions, which involved accuracy, sensitivity, specificity, and the AUC; and (4) sources of funding and the approval of ethics. This study extracted sensitivity and specificity data from each research report, all based on the “optimal threshold” set in the respective original studies. This research did not standardize or adjust for the differences in thresholds among the various studies.

### Bias and Applicability Assessment

#### Overview

We used PROBAST (Prediction Model Risk of Bias Assessment Tool) as the primary instrument to preserve comparability with prior preeclampsia meta-analyses (for detailed information, see Multimedia Appendix 3). Because many included studies predate PROBAST-AI and lack AI-specific reporting (eg, leakage safeguards, hyperparameter tuning, calibration, and thresholds), a full PROBAST-AI assessment would be dominated by underreporting rather than demonstrated bias. The PROBAST [35] was used to assess the risk of bias in the included studies across 4 domains, namely participants, predictors, outcomes, and analysis. Additionally, applicability assessments were conducted for the domains of population, predictors, and outcomes. Two researchers (LL and QZ) independently reviewed the studies, undergoing consistency training based on a preprepared and trialed scoring manual. The discrepancies were resolved through discussion, and if necessary, a third researcher (JW) acted as an adjudicator.

#### Bias Assessment

For all questions within a category, if the answers are “yes” or “possibly,” the category is assessed as low risk. Conversely, if any answer is “no” or “possibly not,” the category is classified as high risk. In cases where there is insufficient information, the category is deemed unclear. The overall risk of bias in the study is determined according to the PROBAST guidelines: (1) if all 4 domains are assessed as low risk, the overall risk of the study is low; (2) if one or more domains are assessed as high risk, the overall risk of the study is high; and (3) if one or more domains are assessed as unclear (and there are no high-risk domains), the overall risk of the study is unclear.

#### Applicability Assessment

The evaluation encompasses 3 categories, including study object, predictor, and outcome. Each category is assessed based on 3 levels of applicability, namely good applicability, poor applicability, and unclear applicability. If all 3 assessments are classified as good, the overall applicability is determined to be good. Conversely, if any one assessment is classified as poor, the overall applicability is deemed poor. In cases where one assessment is unclear while the other two are good, the overall applicability is classified as unclear.

### Statistical Analysis

The methods described in the guidelines for conducting systematic reviews and meta-analyses concerning the performance of prediction models, along with previous meta-analyses of such models, indicate that the concordance index of a model is similar to the AUC [36]. This index indicates the diagnostic or prognostic discrimination ability, categorized as none (AUC≤0.6), poor (0.6<AUC<0.7), moderate (0.7<AUC<0.8), good (0.8<AUC<0.9), or excellent (0.9<AUC<1). Model calibration acts as an indicator of how well the model fits the data by evaluating the alignment between the actual and forecasted results, while also demonstrating the model’s reliability via calibration graphs. Additionally, the diagnostic odds ratio (DOR) is calculated using the following formula:

DOR=PLR/NLR

In this study, we use the positive likelihood ratio (PLR) and the negative likelihood ratio (NLR) to evaluate the predictive performance of our model for preeclampsia. The equations used to calculate PLR and NLR express the frequency of preeclampsia in individuals who are predicted by the model to have preeclampsia compared to those who are predicted not to have preeclampsia:

PLR=Sensitivity/(1-Specificity)

NLR=(1-Sensitivity)/Specificity

Considering the diversity in populations, predictors, and algorithms across the included ML models, our objective was to generalize findings to broader clinical contexts. Therefore, following the recommendation of Borenstein et al [37], we a priori selected the random-effects model for all meta-analyses, irrespective of the magnitude of statistical heterogeneity (*I*^2^). Specifically, we used the more robust Hartung-Knapp-Sidik-Jonkman (HKSJ) method for final pooled estimates and interval calculations to ensure the robustness of statistical inferences [38]. The ML models included in this study exhibited substantial variations in sample size and population characteristics, with the *I*^2^ statistic often approaching 100% in larger samples, potentially limiting their ability to effectively distinguish the actual clinical impact of heterogeneity. Therefore, in addition to reporting the 95% CI for pooled effect sizes, this study further calculated the 95% PI. Unlike CIs, which only reflect the precision of the average effect, PIs estimate the expected range of performance when the model is applied in a new, similar clinical setting in the future. This approach provides a more intuitive assessment of the model’s clinical applicability and transferability [38]. Since the Meta-DiSC software (The developer is the clinical biostatistics team at Ramón y Cajal Hospital) cannot calculate PIs, we used the *meta* package (version 7.0) [39] in R software (R Foundation for Statistical Computing; version 4.4.2) with the HKSJ method to compute 95% PIs for area under the receiver operating characteristic curve (AUROC), sensitivity, and specificity. For AUROC values without reported SEs, we estimated them based on sample size using the Hanley & McNeil [40] method. External validation is regarded as the “gold standard” for assessing the transportability of models. Therefore, a separate evaluation of the performance of models that use external validation is conducted. Subsequently, the 4 predictive models with the highest and lowest values were excluded to conduct a sensitivity analysis aimed at evaluating the impact of outliers on the sensitivity and specificity of the summary. To reduce conceptual heterogeneity and enhance the interpretability of results, stratification is performed along the following dimensions: sample size (less than 2000 and greater than or equal to 2000); data source (electronic medical records; laboratory biomarkers; omics or imaging; mixed); gestational age window (early pregnancy; midpregnancy and late pregnancy or specific gestational weeks); and validation methods (internal validation and external validation); ML models (logistic regression [LR] and nonlogistic regression), followed by more detailed subgroup analysis (LR, extreme gradient boosting [XGBoost], random forest [RF], and support vector machine [SVM]) based on nonlogistic regression; types of predictive variables (demographic information; biological genetic markers; laboratory tests; demographic information and laboratory tests); and the number of predictive variables (less than 10 and greater than or equal to 10). Handling of missing data (extraction and synthesis). For each study, we recorded how missing data were handled and classified methods into 5 categories, namely listwise deletion, single-value imputation (eg, mean and median), multiple imputation, other (eg, random subset iterations), and not reported. When multiple approaches were mentioned, we coded the method used for the primary model. We summarize the overall distribution in the results of “Inclusion of Study Characteristics in the Paper” and discuss implications for comparability and generalizability. Subgroup analyses will be conducted on the included studies to evaluate the performance of ML methods in predicting preeclampsia across different clinical scenarios. Subgroup Analysis discusses the capabilities of different ML algorithms in predicting preeclampsia. Additionally, meta-regression was used to investigate the sources of heterogeneity. Given the extreme heterogeneity (*I*^2^>99%) observed across studies and the lack of standardized threshold reporting (eg, fixed false-positive rates), hierarchical or bivariate models often fail to converge or yield unstable estimates. Therefore, we prioritized univariate random-effects models using the HKSJ adjustment for pooling sensitivity and specificity separately. This method is demonstrated to provide more robust coverage probabilities for CIs in the presence of substantial heterogeneity compared to standard DerSimonian-Laird [41] methods.

## Results

### Literature Screening

**Figure 1:**
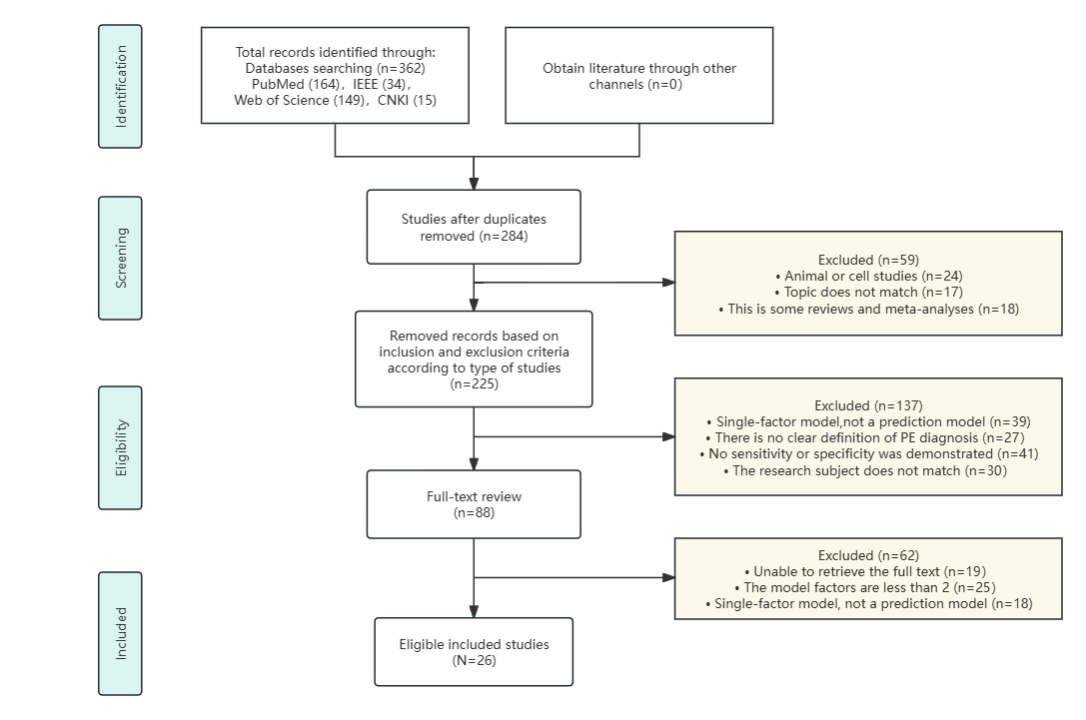
PRISMA (Preferred Reporting Items for Systematic Reviews and Meta-Analyses) flow diagram for study selection. CNKI: China National Knowledge Infrastructure; PE: preeclampsia.

After removing duplicate entries, a total of 284 papers were evaluated. Of these, 284 papers were evaluated through abstract screening, which was subsequently followed by a full-text evaluation of 88 papers. This process culminated in the identification of 26 papers [8-33] that satisfied the overall inclusion criteria. The literature screening procedure and its outcomes are depicted in the related Figure 1.

### Inclusion of Study Characteristics in the Paper

The literature included in this study spans from 2019 to 2025 and consists of 23 English papers [8-10,12-26,28-30,32,33] and 3 Chinese papers [11,27,31]. When a study presented more than 2 models, the top 2 models demonstrating the best performance were selected based on a comprehensive evaluation of metrics, such as AUC, sensitivity, and specificity, culminating in the inclusion of 31 models from 26 papers [8-33]. The data sources for ML predominantly consisted of clinical electronic health records, community research cohorts, and self-administered questionnaires. The overall sample sizes in the studies examined showed considerable variation, fluctuating between 53 and 62,562 cases, while the count of predictors in the ultimate models ranged from 3 to 50. Among all the studies, 20 [8,11,13,14,16-18,20-32] conducted internal validation, while 6 [8,21,24,28,30,32] performed external validation. To assess model performance, the AUC, sensitivity, and specificity emerged as the most frequently used metrics. Among the 26 studies [8-33] reviewed, 5 (19.2%) studies [8,17,25,26,33] were prospective cohort studies, 17 (65.4%) studies [10,11,13-16,18,19,21-24,27-29,31,32] were retrospective cohort studies, 2 (7.7%) studies [9,20] were case-control studies, 1 (3.8%) study [30] was a retrospective case-control study, and 1 (3.8%) study [12]was a multicenter study. Regarding model approaches, of the 31 models included, 3 were LR. Among the remaining 28 models, there were 5 RF, 4 XGBoost, 4 Elastic-net, 3 neural network (NN), 3 SVM, 2 light gradient boosting, 2 AdaBoost, 1 k-nearest neighbor, 1 Naive Bayes, 1 stochastic gradient boosting, 1 CatBoost, and 1 voting classifier. In terms of handling missing data, 8 studies [11,18,22,24-27,29] opted to delete cases with missing data, 7 studies [9,12,14-16,19,23] used mean imputation to address the missing values, 3 studies [13,17,31] used multiple imputation techniques, 1 study [21] implemented random selection of data subsets for multiple iterative analyses, while the remaining 7 studies [8,10,20,28,30,32,33] did not explicitly report the presence of missing values. Such variation limits comparability and external transportability of performance metrics and increases uncertainty around calibration and threshold transfer. The specific details of the models are presented in Table 1.

**Table 1:** Construction of the risk prediction model for preeclampsia.

Literature and modeling method		Model performance									Sample size (modeling; internal validation; external validation)			Missing data							Predictors
		AUC^a^				Sensitivity		Specificity					Quantity (PCS^b^)			Handling method					
**Ansbacher et al** **[8]**																					
	FfNN^c^	0.816				0.533		0.9		30437/10000/20352			—^d^			—			10 predictors: maternal age, maternal weight, maternal height, interpregnancy interval, ethnicity, medical history (such as chronic hypertension, diabetes, etc), uterine artery pulsatility index, mean arterial pressure, placental growth factor, and pregnancy-associated plasma protein-A.		
**Araújo et al** **[9]**																					
	LGB^e^	0.9				0.95		0.79		132/—/—			—			Mean imputation			3 predictors: neutrophil count, mean corpuscular hemoglobin, and aggregate index of systemic inflammation.		
**Chen et al** **[10]**																					
	SVM^f^	0.88				0.87		0.76		166/—/—			—			—			7 predictors: IL-17, IL-21, IL-22, IL-10, transforming growth factor-β, placental alkaline phosphatase, and lysosome-associated membrane protein 3.		
**Chen et al** **[11]**																					
	CB^g^	0.983				0.8881		0.9848		1325/398/—			—			Delete			18 predictors: BMI, systolic blood pressure, diastolic blood pressure, number of pregnancies, mean corpuscular hemoglobin concentration, bacteria (urinalysis), glycocholic acid, high-density lipoprotein, potassium, sodium, phosphorus, uric acid, urine protein, creatinine, direct bilirubin, low-density lipoprotein, gestational age≥34 weeks, and family history of hypertension.		
**Giménez et al** **[12]**											597/—/—			—			Mean imputation			6 predictors: gestational age, history of chronic hypertension, Soluble FMS-like Tyrosine Kinase-1, placental growth factor, N-terminal pro-brain natriuretic peptide, and uric acid.	
	PTB-RF^h^	0.901				0.796		0.91													
	RF^i^	0.941				0.775		0.949													
**Jhee et al** **[13]**																					
	SGB^j^	0.924				0.603		0.991		7704/3302/—			25			Multiple Imputation			14 predictors: systolic blood pressure, serum urea nitrogen, serum creatinine, platelet count, serum potassium level, white blood cell count, serum calcium level, and urinary protein.		
**Kaya et al** **[14]**																					
	XGBoost^k^	0.767				0.6		0.833		53/20/—			—			Mean imputation			8 predictors: maternal age, BMI, smoking status, history of diabetes, history of gestational diabetes, mean arterial pressure, and history of previous preeclampsia.		
**Kovacheva et al** **[15]**											1125/—/—			—			Mean imputation			7 predictors: maternal age, BMI, systolic blood pressure, diastolic blood pressure, uric acid, history of kidney disease, and SBP PRS^m^.	
	LR^l^	0.83				0.85		0.66													
	XGBoost	0.91				0.96		0.44													
**Li et al** **[16]**																					
	XGBoost	0.955				0.789		0.93		3759/191/—			—			Mean imputation			38 predictors: maternal age, BMI, mean blood pressure, abdominal circumference, gravidity, parity, history of preeclampsia, history of previous cesarean section, interpregnancy interval, primipara, multiple gestation, assisted reproductive technology, heart disease, pregestational diabetes, thyroid disease, kidney disease, autoimmune disease, mental illness, uterine fibroids, adenomyosis, uterine malformation, history of epilepsy, family history of hypertension, hemoglobin, white blood cell count, platelet count, creatinine, fasting blood glucose, total cholesterol, high-density lipoprotein, low-density lipoprotein, total protein, albumin, bile acids, uric acid, total bilirubin, direct bilirubin, and gamma-glutamyl transferase.		
**Li et al** **[17]**																					
	VC^n^		0.831			0.77		0.769		3715/929/—						Multiple Imputation			16 predictors: maternal age, height, prepregnancy weight, primiparity, mode of conception, family history, smoking status, history of preeclampsia, history of chronic hypertension, history of chronic kidney disease, history of diabetes, history of systemic lupus erythematosus/antiphospholipid syndrome, mean arterial pressure, uterine artery pulsatility index, pregnancy-associated placental protein a, and placental growth factor.		
**Lv et al** **[18]**																					
	XGBoost		0.963			0.917		0.894		832/208/—			—			Delete			6 predictors: prepregnancy BMI, gravidity, mean arterial pressure, smoking, alpha-fetoprotein, and conception method.		
**Marić et al** **[19]**																					
	EN^o^		0.79			0.452		0.919		5245/—/—			—			Mean imputation			55 predictors: maternal age, height, weight, ethnicity, number of fetuses, mean systolic blood pressure, mean diastolic blood pressure, maximum systolic blood pressure, maximum diastolic blood pressure, history of preeclampsia, chronic hypertension, type 1 and type 1 diabetes, gestational diabetes, obesity, assisted reproductive technology, diagnosis of autoimmune diseases, kidney disease, anemia, antiphospholipid syndrome, sexually transmitted diseases, hyperemesis gravidarum, headache, migraine, poor obstetric history, high-risk pregnancy, protein and glucose in urine, platelet count, red blood cells, white blood cells, creatinine, hemoglobin, hematocrit, monocytes, lymphocytes, eosinophils, neutrophils, basophils, Rh blood type, gastric acid, rubella, chickenpox, hepatitis B virus, syphilis, gonorrhea, aspirin, nifedipine, aldomet, labetalol, insulin, glyburide, prednisone, azathioprine, Plaquenil, heparin, levothyroxine, doxylamine, and acyclovir.		
**Melinte-Popescu et al** **[20]**																					
	NB^p^		0.98			0.963		0.964		163/70/—			—			—			14 predictors: age, BMI, smoking status, interpregnancy interval, use of assisted reproductive technology, pregestational diabetes, chronic hypertension, history of kidney disease, personal or family history of preeclampsia, placental growth factor, pregnancy-associated plasma protein A, placental protein 13, uterine artery pulsatility index, and mean arterial pressure.		
**Munchel et al** **[21]**																					
	AB^q^		0.964			0.88		0.92		113/11/448			—			Randomly select a subset of data for multiple iterative analyses.			49 predictors circulating transcripts in blood: immunomodulatory, fetal development, angiogenesis, and extracellular matrix remodeling.		
**Roque et al** **[22]**																					
	LR		0.976			0.9		0.951		35706/8927/—			—			Delete			11 predictors: platelet count, white blood cell count, lymphocyte percentage, monocyte percentage, red blood cell count, red cell distribution width, platelet distribution width, band neutrophil percentage, red cell distribution width, hematocrit, and maternal age.		
**Sandström** **et al** **[23]**																					
	LR		0.67			0.282		0.9		62562/6256/—			—			Mean imputation			36 predictors: gestational age at first visit, maternal age, BMI, mean arterial pressure, capillary blood glucose level, urine protein, hemoglobin level, history of miscarriage, history of ectopic pregnancy, history of infertility treatment, family status, country of birth, smoking history, smoking status at registration, use of snuff in the first trimester of pregnancy, use of snuff during pregnancy, alcohol consumption in the 3 months before registration, alcohol consumption habits at the time of pregnancy registration, family history of preeclampsia, infertility, family history of hypertension, previous diabetes, chronic hypertension, chronic kidney disease, cardiovascular disease, endocrine disease, history of thrombosis, history of mental illness, history of epilepsy, Crohn/ulcerative colitis, lung disease or asthma, hepatitis, gynecological disease or surgery, recurrent urinary tract infections, and blood type.		
**Sufriyana et al** **[24]**																					
	RF		0.86			0.7		0.89		23201/20975/GEV^r^:1322, TEV^s^: 90			301			Delete			13 predictors: age, family role, parity, type of work, infectious diseases, endocrine, nutritional and metabolic diseases, circulatory system diseases, immune-related diseases, ophthalmic diseases, urogenital diseases, skin and subcutaneous tissue–related diseases, breast-related diseases, digestive system–related diseases, and skin-related diseases.		
**Tiruneh et al** **[25]**																					
	RF		0.84			0.76		0.79		33767/14475/—			66			Delete			13 predictors: maternal age, ethnicity, prepregnancy/early pregnancy BMI, history of preeclampsia in previous pregnancies, primiparity, history of gestational diabetes, pre-existing hypertension, diabetes, family history of hypertension and diabetes, family history of preeclampsia, renal disease, smoking history, and polycystic ovary syndrome.		
**Torres et al** **[26]**											1068/914/—			78			Delete			13 predictors: placental growth factor, mean arterial pressure, uterine artery pulsatility index, BMI, antiphospholipid syndrome, previous preeclampsia, previous diabetes, smoking status, natural conception, Other drug use (such as cocaine and heroin), systemic lupus erythematosus, chronic hypertension, and maternal age.	
	all-EN		0.778			0.501		0.9													
	EPE-EN^t^		0.963			0.882		0.9													
	PPE-EN^u^		0.897			0.765		0.9													
**Wang et al** **[27]**																					
	KNN^v^		0.9			0.7142		0.926		516/172/—			—			Delete			7 predictors: urine protein, urine conductivity, alkaline phosphatase, serum uric acid, lactate dehydrogenase, mean corpuscular hemoglobin concentration, and amylase.		
**Wang et al** **[28]**																					
	AB		0.8775			0.7271		0.9		25709/77713/1760			—			—			20 predictors: maternal age, maternal BMI, regularity of maternal menstrual cycle, vomiting and nausea during pregnancy, previous miscarriages, preterm births, history of hypertension during pregnancy, hypertension, diabetes, chronic hypertension, history of drug allergies, maternal smoking history, previous delivery history, nutritional status during pregnancy, maternal ethnic background, history of hypertension, history of diabetes, glycated hemoglobin, and albumin.		
**Xue et al** **[29]**																					
	SVM			0.93	0.67		0.999		800/160/—			—			Delete			50 predictors: diabetes mellitus, thrombotic diseases, systemic lupus erythematosus, antiphospholipid syndrome, renal diseases, assisted reproductive technology, obstructive sleep apnea syndrome, prepregnancy BMI>30 kg/m², age>35 years, multiple pregnancy, primipara, history of eclampsia or preeclampsia, Albumin, Alanine aminotransferase, Aspartate aminotransferase, Alkaline phosphatase, Complement C1q, Calcium, Creatinine, C-reactive protein, Cystatin C, Gamma-glutamyl transferase, Globulin, Triglycerides, Total cholesterol, High-density lipoprotein cholesterol, Low-density lipoprotein cholesterol, Lipoprotein(a), Apolipoprotein A1, Apolipoprotein B, Small dense low-density lipoprotein, Total protein, Total bile acid, Total bilirubin, Direct bilirubin, Uric acid, Urea, Phosphorus, Absolute Lymphocyte count, Absolute neutrophil count, Platelet count, NEU/LYM ratio, PLT/LYM ratio, Prothrombin time, Prothrombin activity, Activated partial thromboplastin time, Fibrinogen, D-Dimer, Fibrin degradation products, Thrombin time.			
**Yu et al** **[30]**																					
	RF			0.96	0.87		0.91		404/1384/899			—			—			12 predictors: maternal age, BMI, parity, medical history (chronic hypertension, preeclampsia, systemic lupus erythematosus, antiphospholipid syndrome), mode of conception; cfDNA profile indicators: Fos-related antigen 2 (FOSL2), calcium/calmodulin-dependent protein kinase kinase 2 (CAMKK2), G1/S-specific cyclin-D1 (CCND1), Inositol 1,4,5-trisphosphate receptor type 1 (ITPR1), Protein kinase A catalytic subunit beta (PRKACB), Protein Wnt-7b (WNT7B), Voltage-dependent L-type calcium channel subunit beta-2(CACNB2), Nuclear respiratory factor 1 (NRF1), Fms-related tyrosine kinase 3 ligand (FLT3LG), Epidermal growth factor (EGF).			
**Zheng et al** **[31]**																					
	LGB			0.964	0.849		0.927		1609/483/—			—			Multiple imputation			12 predictors: urine specific gravity, uric acid, mean corpuscular hemoglobin concentration, globulin, platelet distribution width, potassium ion, age, family history of hypertension, systolic blood pressure, diastolic blood pressure, pulse, and gestational age≥34 weeks.			
**Zhou et al** **[32]**											432/197/288			—			—			19 predictors: mRNA markers: Albumin, Fibrinogen Alpha Chain, Leptin, Insulin-Like Growth Factor Binding Protein 5, Alpha-1 Antitrypsin, S100 Calcium Binding Protein A9, Apolipoprotein A1, Thyroid Stimulating Hormone Beta Subunit, miRNA markers: MIR130A, MIR144, MIR19B1, MIR215, MIR376C, MIR27A, MIR106A, MIR33A, Inc ENA markers**:** Macrophage Migration Inhibitory Factor, Assisted Reproductive Technology, Mean Arterial Pressure.	
	AvNN^w^			0.91	0.63		0.93														
	SVM			0.93	0.47		0.99														
**Zhou et al** **[33]**																					
	CNN^x^			0.883	0.722		0.934		1138/—/—			—			—			8 predictors: Retinal fundus image score, Prepregnancy BMI, maternal age, chronic hypertension, diabetes, history of gestational hypertension or preeclampsia, assisted reproductive technology, and autoimmune diseases.			

^a^AUC: area under the curve.

^b^PCS: pieces.

^c^FfNN: feed-forward neural network.

^d^not reported.

^e^LGB: light gradient boosting.

^f^SVM: support vector machine.

^g^CB: CatBoost.

^h^PTB-RF: Premature birth - Random Forest.

^i^RF: random forest.

^f^KNN: k-nearest neighbor.

^j^SGB: stochastic gradient boosting.

^k^XGBoost: extreme gradient boosting.

^l^LR: logistic regression.

^m^SBP PRS: systolic blood pressure polygenic risk score.

^n^VC: Voting Classifier.

^o^EN: Elastic-net.

^p^NB: Naive Bayes.

^q^AB: AdaBoost.

^r^GEV: geographic external validation

^s^TEV: temporal external validation

^t^EPE-EN: early onset of preeclampsia Elastic-net.

^u^PPE-EN: Premature birth of preeclampsia Elastic-net.

^v^KNN: k-nearest neighbor.

^w^AvNN: Average Neural Network.

^x^CNN: Convolutional Neural Networks.

### Research Quality

We evaluated the potential for bias and the relevance of the prediction models based on the PROBAST checklist, examining a total of 26 [8-33] studies. Among these, 3 (12%) studies [9,11,16] in the participant domain exhibited unclear risk of bias, primarily due to their case-control design, which is inherently associated with a higher risk of selection bias. In the predictor domain, 1 (4%) study [21] was identified as having unclear risk of bias because it used C-RNA transcriptome assays that depend on transcriptome enrichment and high-throughput sequencing, methods that are not typically used in routine clinical testing. In the analysis of bias domains, 8 (31%) studies [9,10,14,16,21,29,32,33] demonstrated unclear risk of bias, mainly due to insufficient sample sizes, unclear methodologies for addressing missing data, and uncertainties regarding the management of overfitting risks. Furthermore, 1 (4%) study [22] was classified with a high risk of bias as all data were sourced from a single hospital, despite the volume of data, failing to represent a multicenter or stratified analysis. Overall, the bias risk was determined to be unclear for 9 (35%) studies [9-11,14,16,21,29,32,33]. The applicability ratings were moderate for 4 (15%) studies [10,11,21,33], high for 1（4%) study [22], and low for the remaining studies [8,9,12-20,23-32], as detailed in Table 2. For the remaining details, see Table S2 in the Multimedia Appendix 2. 

**Table 2:** Risk of bias and applicability assessment using PROBAST (Prediction Model Risk of Bias Assessment Tool).

Study and year	ROB^a^					Overall bias rating		Overall applicability rating		External validation
	Participants	Predictors	Outcome	Analysis						
Ansbacher et al [8], 2022	Low	Low	Low	Low	Low		Low		Yes	
Araújo et al [9], 2024	Unclear	Low	Low	Unclear	Unclear		Low		No	
Chen et al [10], 2022	Low	Low	Low	Unclear	Unclear		Unclear		No	
Chen et al [11], 2023	Unclear	Low	Low	Low	Unclear		Unclear		No	
Garrido-Giménez et al [12], 2023	Low	Low	Low	Low	Low		Low		No	
Jhee et al [13], 2019	Low	Low	Low	Low	Low		Low		No	
Kaya et al [14], 2024	Low	Low	Low	Unclear	Unclear		Low		No	
Kovacheva et al [15], 2023	Low	Low	Low	Low	Low		Low		No	
Li et al [16], 2021	Unclear	Low	Low	Unclear	Unclear		Low		No	
Li et al [17], 2024	Low	Low	Low	Low	Low		Low		No	
Lv et al [18], 2025	Low	Low	Low	Low	Low		Low		No	
Marić et al [19], 2020	Low	Low	Low	Low	Low		Low		No	
Melinte-Popescu et al [20], 2023	Low	Low	Low	Low	Low		Low		No	
Munchel et al [21], 2020	Low	Unclear	Low	Unclear	Unclear		Unclear		Yes	
Roque et al [22], 2024	Low	Low	Low	High	Low		High		No	
Sandström et al [23], 2019	Low	Low	Low	Low	Low		Low		No	
Sufriyana et al [24], 2020	Low	Low	Low	Low	Low		Low		Yes	
Tiruneh et al [25], 2024	Low	Low	Low	Low	Low		Low		No	
Torres et al [26], 2024	Low	Low	Low	Low	Low		Low		No	
Wang et al [27], 2022	Low	Low	Low	Low	Low		Low		No	
Wang et al [28], 2024	Low	Low	Low	Low	Low		Low		Yes	
Xue et al [29], 2023	Low	Low	Low	Unclear	Unclear		Low		No	
Yu et al [30], 2024	Low	Low	Low	Low	Low		Low		Yes	
Zheng et al [31], 2021	Low	Low	Low	Low	Low		Low		No	
Zhou et al [32], 2024	Low	Low	Low	Unclear	Unclear		Low		Yes	
Zhou et al [33], 2023	Low	Low	Low	Unclear	Unclear		Unclear		No	

^a^ROB: risk of bias.

### The Performance of ML Models in Preeclampsia Prediction

**Figure 2:**
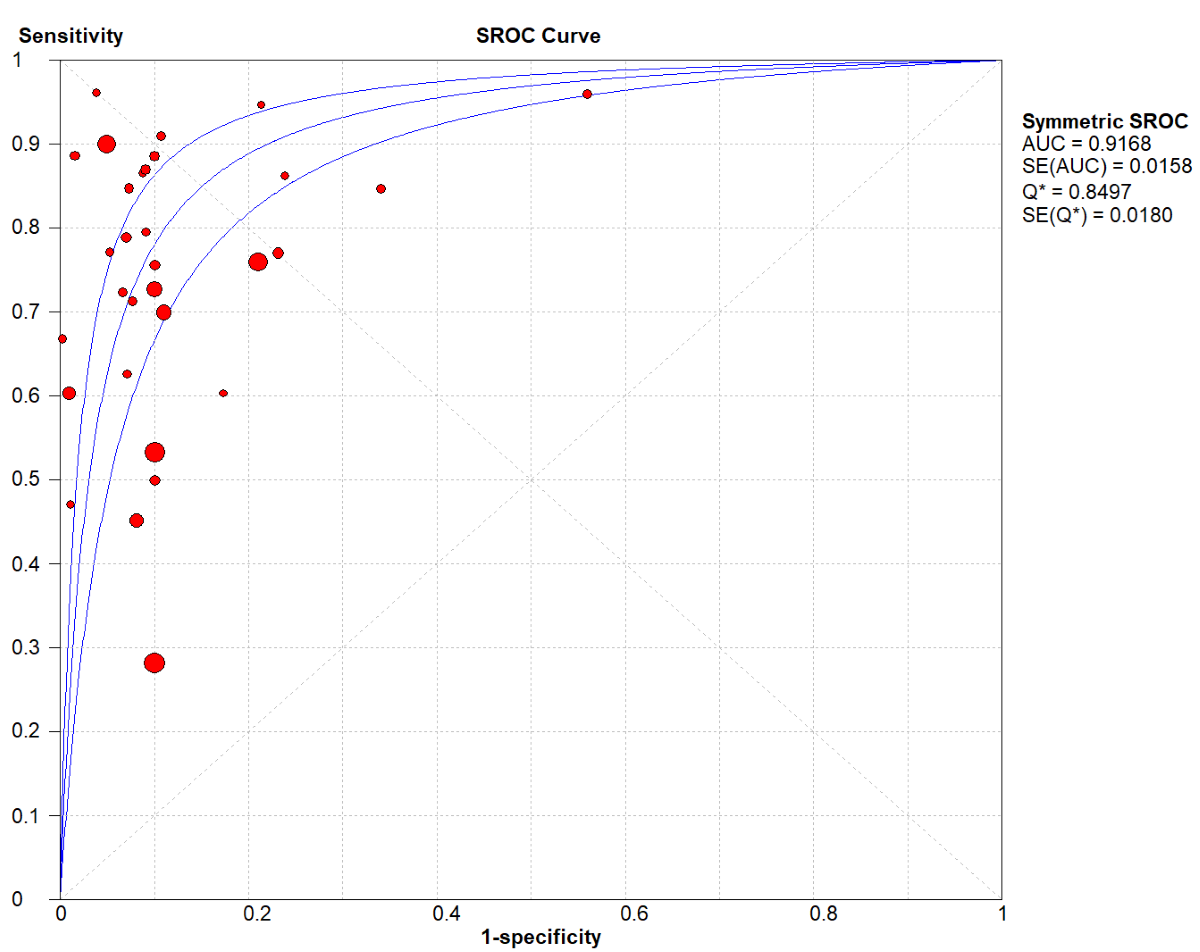
Summary Receiver Operating Characteristic (SROC) plot illustrating the dispersion of study results. AUC: area under the curve; SROC: Summary Receiver Operating Characteristic.

**Figure 3:**
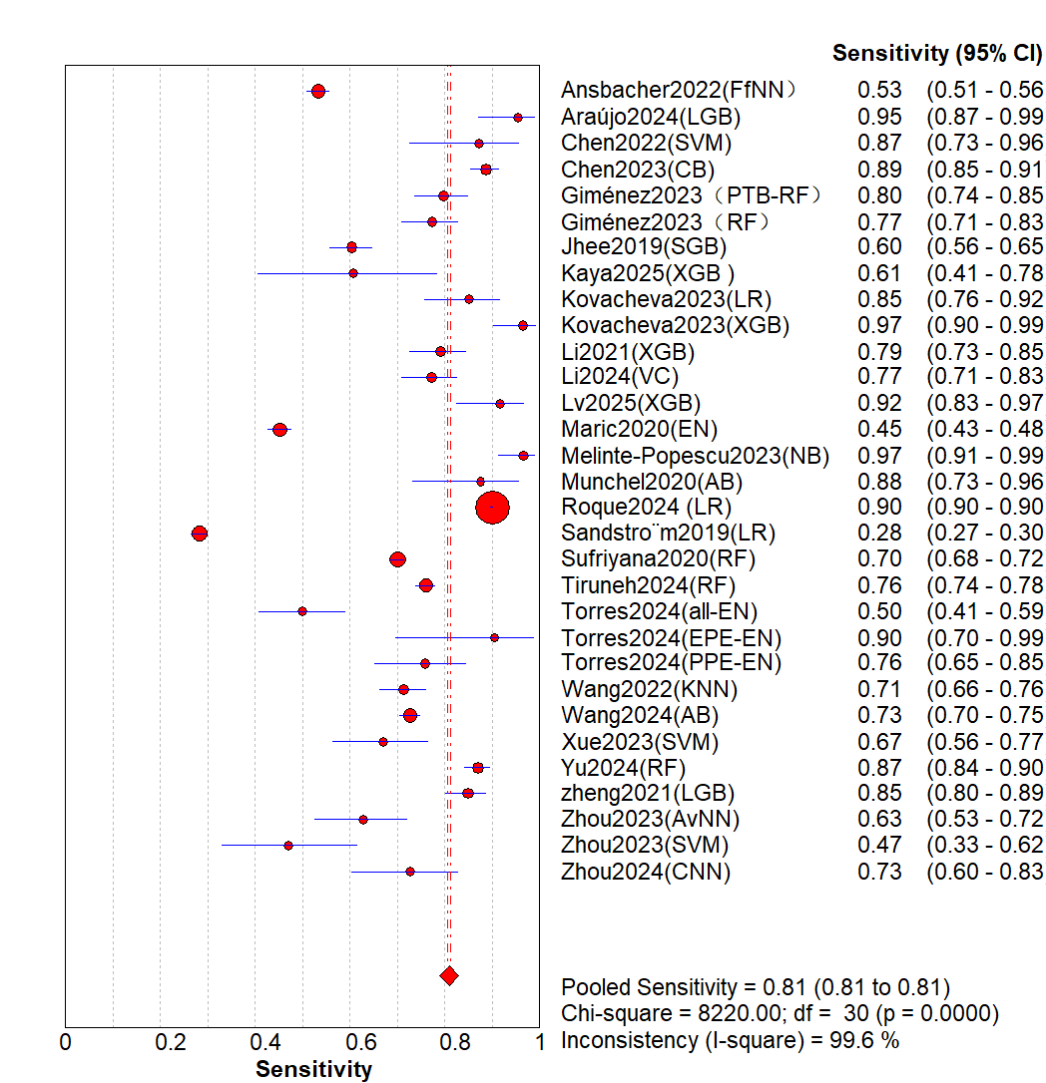
Overall sensitivity of machine learning models for the prediction of preeclampsia [13-17, 19-39].

**Figure 4:**
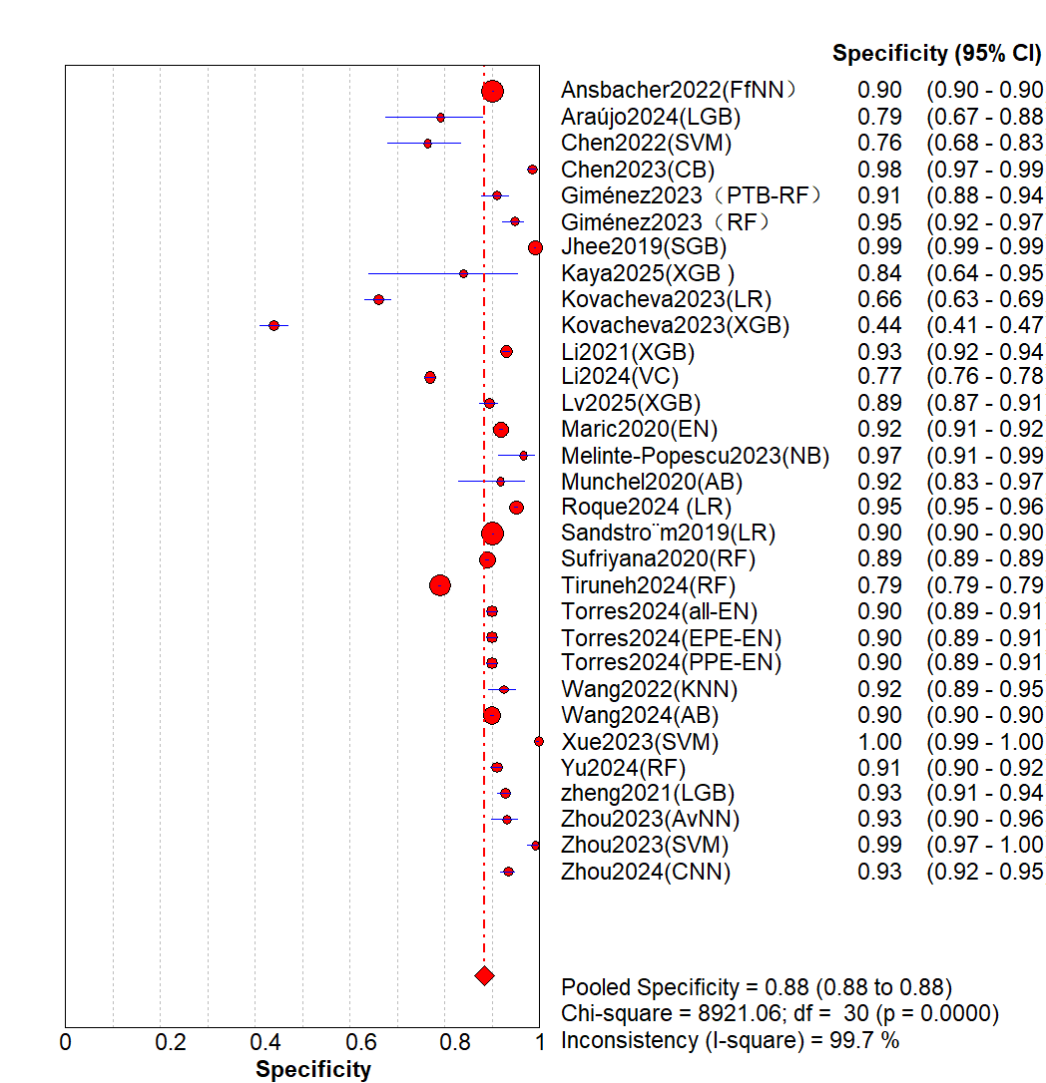
Overall summary specificity of machine learning for the prediction of preeclampsia. [13-17, 19-39].

A total of 26 (31 models) studies [8-33] were included. While the pooled estimates demonstrated high average discriminative potential of ML models, substantial between-study heterogeneity was observed, indicating significant context-dependency of model performance. The overall pooled AUROC was 0.91 (95% CI 0.87-0.92; Figure 2). However, its 95% PI ranged from 0.75 to 1.00, suggesting that AUC might decrease to 0.75 in some external validation settings. The pooled sensitivity was 0.81 (95% CI 0.70-0.83; *P*<.001; *I*^2^=99.6%) In the Figure 3 [8-33], the first author of each study is listed along the Y-axis, the circles represent the point estimates of sensitivity for each model, with the size of the circles being proportional to the weight of the study; the horizontal lines indicate their 95% CIs. The letter Q represents the intersection point of the SROC curve with the inverse diagonal line where “Sensitivity = Specificity.” The diamonds represent the aggregated sensitivity estimates of the models, with their width corresponding to the 95% CI of the aggregated values. The vertical red dashed line represents the 95% CI of the pooled sensitivity. However, this only represents an average level; the wide 95% PI of 0.32-0.96] reveals potential clinical risks. In certain specific studies or future applications, the sensitivity may be as low as 32%, indicating a substantial risk of missed diagnoses. Similarly, although the pooled specificity was 0.88 (95% CI 0.84-0.94; *P*<.001; *I*^2^=99.7%; Figure 4 [8-33]), its PI across different contexts was 0.49-0.99, demonstrating a similar lack of consistency in specificity. The other summary metrics were as follows: DOR was 37.67 (95% CI 23.46-60.48); PLR was 8.52 (95% CI 6.43-11.29); NLR was 0.24 (95% CI 0.18-0.34). Additionally, we calculated the Spearman correlation coefficient between the log of sensitivity and the log of (1-specificity), which yielded a result of 0.254 (*P*=.17), indicating no significant threshold effect in the included studies. This suggests that the observed high heterogeneity (as well as the broad PIs mentioned above) primarily stems from nonthreshold factors (such as differences in predictor selection or population characteristics), rather than merely from variations in cutoff value selection.

### Performance Analysis of External Validation Models

**Figure 5:**
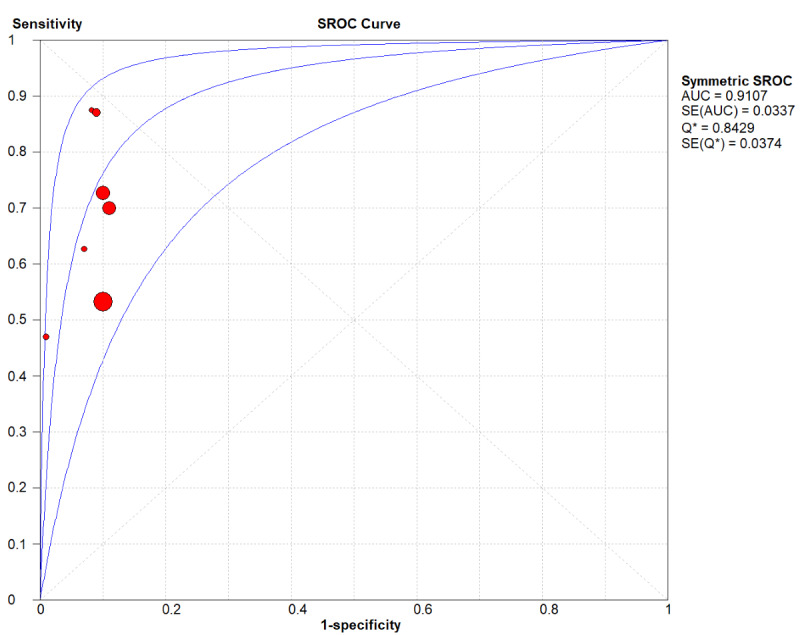
Summary Receiver Operating Characteristic (SROC) plot for external validation models. AUC: area under the curve; SROC: Summary Receiver Operating Characteristic.

**Figure 6:**
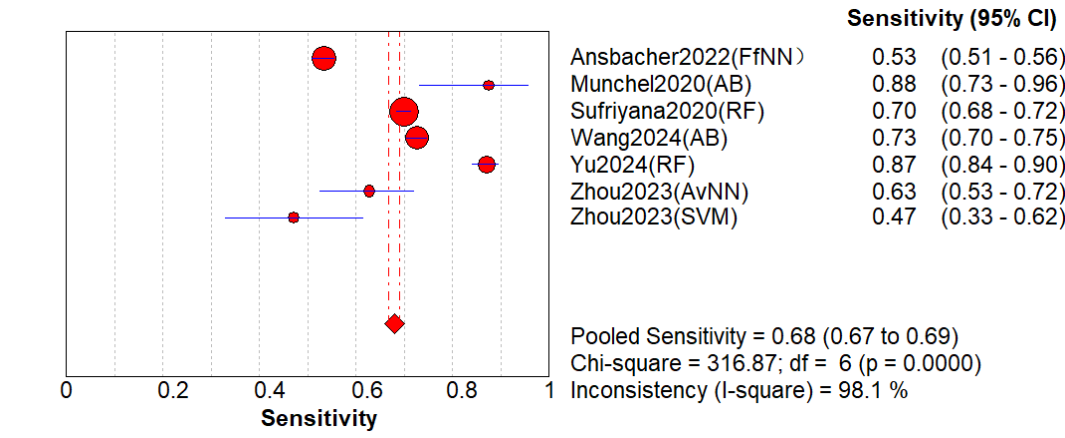
Summary sensitivity of machine learning models for predicting preeclampsia based on external validation [13,27,30,34,36,38].

**Figure 7:**
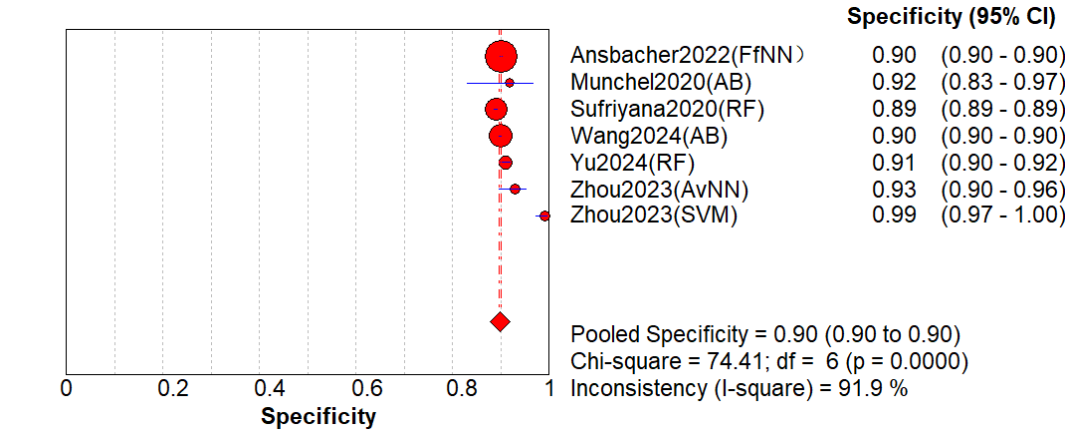
Overall summary specificity of machine learning for predicting preeclampsia [13,27,30,34,36,38].

A total of 6 (comprising 7 models) studies [8,17,24,28,30,32] underwent external validation. The analysis revealed that when applied to independent external populations, the models exhibited performance decline with persistent high heterogeneity. Specifically, the pooled AUC was 0.91 (95% CI 0.85-0.95; Figure 5). However, its 95% PI was 0.76-1.00, indicating that the model’s discriminative ability might be suboptimal in certain external settings. The pooled sensitivity significantly decreased to 0.68 (95% CI 0.54-0.83; *P*<.001; *I*^2^=99.6%; Figure 6 [8,21,24,28,30,32]), with a 95% PI of 0.25-0.94. The lower limit of 0.25 indicates that in the worst-case external validation scenario, the model may miss 75% (23/31) of patients, posing an extremely high risk of missed diagnosis. The pooled specificity was 0.90 (95% CI 0.86-0.96; *P*<.001; *I*^2^=99.7%; Figure 7 [8,21,24,28,30,32]), with a 95% PI of 0.62-0.99. Other indicators included: DOR of 28.21 (95% CI 18.10-43.98; *I*^2^=97.6%); PLR of 7.51; NLR of 0.32. The decrease in sensitivity (from 0.81 in the primary analysis to 0.68) and the extremely low limit of the PI (0.25) strongly confirmed the limited transportability of the model across populations, indicating that direct clinical application requires extreme caution.

### Sensitivity Analysis

**Figure 8:**
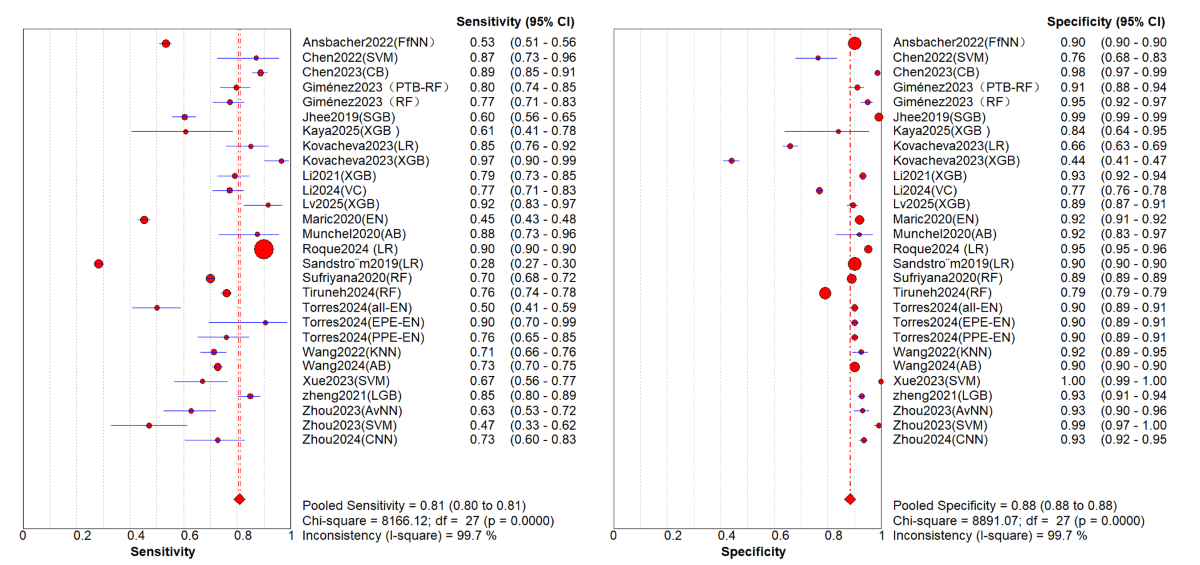
Forest plots of diagnostic performance [13-17, 19-39].

After conducting a sensitivity analysis excluding case-control studies in a leave-one-domain-out with 4 (15%) models, the overall summary AUROC is 0.9109 (95% CI 0.8642-0.9390). The summary sensitivity estimate derived from the random-effects meta-analysis is 0.81 (95% CI 0.70-0.83; *P*<.001; *I*^2^=99.7%), and the summary specificity is 0.88 (95% CI 0.84-0.94; *P*<.001; *I*^2^=99.7%), as detailed in Figure 8 [8-33]. Consequently, it was concluded that the pooled estimates remained unaffected by the exclusion of outlier values. With an AUC>0.8, the model demonstrated good discriminative ability, but an *I*^2^>75% indicated substantial heterogeneity within most subgroups. To address this issue and gain deeper insights, we undertook a subgroup analysis to investigate the potential sources of this heterogeneity across the studies that were included in our review. Accordingly, we do not interpret a single pooled estimate as “average clinical performance” and instead prioritize subgroup results. In addition, to eliminate the impact of multiple models (derived from the same population) within a single study on statistical independence (unit-of-analysis error), we conducted additional sensitivity analyses by retaining only the model with the highest AUROC from each study (N=26). The results showed that the pooled sensitivity after deduplication was 0.81 (95% CI 0.73-0.87), specificity was 0.88 (95% CI 0.83-0.91), and AUROC was 0.90 (95% CI 0.87-0.93). The above results were highly consistent with the primary analysis (N=31), with no significant differences observed in the CIs, indicating that incorporating different models from the same study did not lead to inflated results or underestimated variance. Therefore, we retained all models in the primary analysis to demonstrate the performance differences among various predictor combinations.

### Subgroup Analysis

The comparative results of the subgroup analysis on preeclampsia prediction performance are presented in Table 3; types of ML models, forest plots are shown in Figures S1-S22 in Multimedia Appendix 2. The comparison between subgroups was determined by examining whether the 95% CI of the AUC overlapped. Nonoverlapping intervals indicated statistical significance while overlapping intervals indicated no statistical significance. Data were derived from electronic health records, high-throughput omics, and hybrid sources. Subgroup analysis indicated that models based on hybrid data demonstrated superior performance, followed by those using electronic health records and high-throughput omics. However, considerable heterogeneity was observed, and the 95% CIs extensively overlapped across the 3 data types, suggesting no statistically significant differences among them. The “pregnancy window” refers to the index timing window during which predictors were collected or model discrimination was performed. Models constructed using third-trimester data showed better performance with low heterogeneity. Nonetheless, overlapping 95% CIs across models indicated no statistically significant differences among pregnancy window subgroups. Regarding validation strategies, internally validated models outperformed externally validated ones, albeit with high heterogeneity. Subgroup analysis revealed overlapping 95% CIs between the 2 validation types, implying that the difference was not statistically significant. Regarding sample size, the subgroup analysis results showed that models with smaller sample sizes outperformed those with larger sample sizes, exhibiting lower heterogeneity. However, since the 95% CI overlapped, the differences between sample size subgroups were not statistically significant. Regarding the adopted model, nonlogistic regression prediction models outperformed logistic regression prediction models. Further analysis was conducted on nonlogistic regression models with 3 or more instances in each model category, revealing that neural networks exhibited the best predictive performance with an AUC of 0.9966 (95% CI 0.9772-1.0000) and the lowest heterogeneity. The difference in model performance was statistically significant when compared to elastic net models, but not statistically significant when compared to other models. Regarding the type of predictive variables, prediction models constructed solely using laboratory test indicators achieved the highest predictive performance with an AUC of 0.9463 (95% CI 0.9097-0.9820) and the lowest heterogeneity. Nevertheless, when compared to models built with alternative indicators, the difference in performance was not statistically significant. For the number of predictor variables used in model building, models with 10 or more variables exhibited higher predictive performance with an AUC of 0.9204 (95% CI 0.8671-0.9737), but the difference was not statistically significant compared to models with fewer than 10 variables.

**Table 3:** Subgroup analysis results.

Grouping							Number of prediction models (PCS^a^)		AUC^b^ (95% CI)		*I*^2^ (%)		*P* value
Entire study							31		0.9168 (0.891-0.950)		99.6		<.001
**Sample size**													
	<2000					16		0.9361 (0.9079-0.9643)		90.9		<.001	
	≥2000					15		0.9109 (0.8501-0.9717)		99.8		<.001	
**Data source**													
		Mixed				14		0.9154 (0.8713-0.9595)		99.6		<.001	
		EHR^c^				12		0.9126 (0.8430-0.982)		99.4		<.001	
		Omics				4		0.9406 (0.8898-0.9914)		95.3		<.001	
**Pregnancy window**													
				Early			10		0.9406 (0.7853-1.0000)		95.2		<.001
				Mid			4		0.9304 (0.8965-0.9643)		77.2		.004
				Late			3		0.9665 (0.9314-1.0000)		71.4		.03
				Specific			14		0.9138 (0.8805-0.9471)		99.1		<.001
**Machine learning model**													
			Logistic regression				3		0.9044 (0.6857-1.0000)		100.0		<.001
			Nonlogistic regression				28		0.9171 (0.8871-0.9471)		97.6		<.001
					RF^d^	5		0.8917 (0.7950-0.9884)		95.8		<.001	
					SVM^e^	3		0.9068 (0.7623-1.0000)		88.2		<.001	
					XGBoost^f^	4		0.9177 (0.8500-0.9854)		89.9		<.001	
					EN^g^	4		0.9419 (0.9125-0.9713)		93.9		<.001	
					NN^h^	3		0.9966 (0.9772-1.0000)		84.7		.001	
**Predictor variable type**													
			Demographic information				10		0.8754 (0.8315-0.9193)		99.4		<.001
			Biological genetic marker				3		0.9300 (0.8375-1.0000)		96.8		<.001
			Demographic information and laboratory tests				13		0.9275 (0.8665-0.9885)		98.4		<.001
			Laboratory testing				5		0.9463 (0.9097-0.9820)		95.8		<.001
**Number of predictor variables**													
			<10				10		0.9124 (0.8855-0.9393)		86.6		<.001
			≥10				21		0.9196 (0.8665-0.9727)		99.8		<.001

^a^PCS: piece.

^b^AUC: area under the curve.

^c^EHR: electronic health record.

^d^RF: random forest.

^e^SVM: support vector machine.

^f^XGBoost: extreme gradient boosting.

^g^EN: elastic network.

^h^NN: neural network.

### Meta-Regression Analysis

Due to the significant heterogeneity observed among the studies, a meta-regression analysis was conducted. The meta-analysis focused on various factors, including sample size, country of publication, type of ML model, year of publication, study design, study quality, and predictors, as detailed in Table 4. Variables were systematically removed based on the magnitude of their *P* values, and separate meta-regression analyses were performed for each variable. The results indicated that the source of heterogeneity among the studies was primarily associated with the research quality, as illustrated in Table 5.

**Table 4:** Meta-regression analysis.

Variable	β coefficient (SE)	*P* value	RDOR^a^(95% CI)
Constant	3.547 (1.3356)	.01	—^b^
Sample size	1.075 (0.5388)	.06	0.34 (0.11-1.05)
Country	–0.322 (0.4741)	.50	0.72 (0.27-1.94)
ML^c^ method	0.588 (0.7387)	.43	1.80 (0.39-8.37)
Year	0.007 (0.4578)	.99	1.01 (0.39-2.61)
Design	–1.435 (0.8047)	.09	0.24 (0.04-1.27)
Quality	0.672 (0.4076)	.11	1.96 (0.84-4.57)
Predictive	0.773 (0.6075)	.22	2.17 (0.61-7.67)
Validation type	–0.318 (0.4797)	.51	0.73 (0.27-1.97)

^a^RDOR: relative diagnostic odds ratio.

^b^Not applicable.

^c^ML: machine learning.

**Table 5:** Meta-regression analysis after excluding *P* values from largest to smallest.

Variable	β coefficient (SE)	*P* value	RDOR^a^ (95% CI)
Constant	2.398 (0.5879)	<.001	—^b^
Quality	0.800 (0.3951)	.05	2.23 (0.99-5.00)

^a^RDOR: relative diagnostic odds ratio.

^b^Not applicable.

## Discussion

### Principal Findings

This systematic review identified 31 ML models for preeclampsia prediction. Our primary finding highlights a critical paradox. While models demonstrate high average discriminative potential (pooled AUROC 0.91), they exhibit extreme heterogeneity (*I*^2^>99%) and limited transportability. The wide 95% PI for sensitivity (0.32-0.96) warns that a model performing perfectly in development may miss nearly 70% of cases when applied to a new population. This “context dependence” is further confirmed by the performance drop in external validation studies (pooled sensitivity of 0.68), suggesting that current high AUROCs largely reflect internal fit rather than universal clinical effectiveness.

To investigate the sources contributing to this heterogeneity (as well as the wide PIs), our subgroup analysis revealed several key factors. In the subgroup analysis of all 31 models, we observed that their predictive performance was better when the sample size was small (less than 2000 cases), which contradicts the conventional understanding that “larger sample sizes lead to better predictive performance” [42]. The analysis may be significantly influenced by confounding factors, such as study design (eg, case-control studies) and research type—especially considering the very high AUC of the elastic net (AUC=0.963 for Torres et al [26]; AUC=0.96 for Yu et al [30]). Therefore, careful discernment is required, and one should not hastily interpret this as indicating superior predictive performance of models with smaller sample sizes. Regarding predictor types, laboratory test indicators exhibit superior predictive performance, as the core pathological mechanisms of preeclampsia include placental perfusion disorders, endothelial dysfunction, oxidative stress, and inflammatory responses [43]. Laboratory indicators can directly reflect pathological states, while demographic information provides only indirect risk assessments.

Among the ML models analyzed in this study, including RF, SVM, NN, and Elastic-net, the NN model demonstrated the highest predictive performance (AUC=0.99, 95% CI 0.98-1.00), surpassing traditional ML methods, such as LR, RF, and extreme gradient boosting. This analysis may be attributed to the complex etiology of preeclampsia, a pregnancy complication characterized by multiple pathological processes. The intricate, multidimensional interactions inherent in preeclampsia are challenging to capture comprehensively using linear models. In contrast, NN models are well-equipped to model nonlinear relationships and higher-order variable interactions, which more accurately reflect the pathological characteristics of preeclampsia [44]. Compared to traditional methods, NN can automatically extract features and assign weights to input variables without the need for extensive manual variable screening, demonstrating particular advantages in handling high-dimensional data [45]. Moreover, NN models can integrate multisource heterogeneous data, such as demographic information, laboratory indicators, and biological genetic markers, thereby adapting to the increasingly complex trends in clinical data.

Higher predictive performance is observed when the number of predictors is equal to or greater than 10. This indicates that using a greater number of predictors helps to more comprehensively reflect disease status, significantly enhancing the model’s predictive performance. This is especially true for nonlinear algorithms, which are better equipped to capture interaction effects and underlying patterns.

Nonstandardized handling of missing data means that AUC; concordance index and calibration may not be directly comparable across studies; in particular, listwise deletion or simple imputation combined with restricted case-mix and threshold tuning can inflate discrimination and understate uncertainty. We therefore recommend at minimum (1) transparent reporting of missingness (overall and by variable) and the primary imputation strategy; (2) preferential use of multiple imputation or model-based methods, with minimal recalibration (slope and Brier) and decision-curve analysis during external validation; and (3) reporting confusion matrices under fixed thresholds and top-N% triage plus subgroup robustness (GA window; outcome definitions and sites) to enhance interpretability for clinical and digital health use.

### Strengths and Limitations

First, regarding methodological rigor and transparency, we strictly adhered to the PRISMA guidelines for reporting, and the research protocol has been preregistered in the international prospective systematic review registry PROSPERO (CRD420251005830). This ensures that the research objectives and methods are predetermined, thereby minimizing reporting bias. Second, concerning the comprehensiveness of the literature search, our search strategy exhibits significant interdisciplinary characteristics. We not only searched mainstream medical databases such as PubMed and CNKI, but also included IEEE Xplore and Web of Science to ensure a comprehensive capture of ML models published in the fields of engineering technology and computer science. This is critical for a topic that bridges clinical medicine and artificial intelligence, avoiding potential omissions of models that might occur if only medical databases were searched. Third, regarding the reliability of data processing, the entire process of literature screening and data extraction in this study was conducted independently by 2 researchers, with any discrepancies resolved through discussion or by involving a third researcher as an adjudicator. This “dual review” process is considered the gold standard for systematic reviews, ensuring the accuracy of data extraction. Fourth, in terms of the professionalism of quality assessment, we used the PROBAST tool, which is currently recommended by international authorities and specifically designed for predictive model research, rather than traditional diagnostic test evaluation tools, such as QUADAS-2 (Whiting and colleagues [46]). PROBAST enables us to thoroughly assess the risk of bias and applicability of the models across 4 key domains, including participants, predictive factors, outcomes, and analysis, which is more in-depth and relevant than previous reviews. Finally, regarding the prudence of analysis, this study recognizes the common pitfall of “performance overestimation” in meta-analyses of predictive models. Therefore, we clearly identified models lacking external validation and conducted an independent meta-analysis of studies that reported external validation. This approach allowed us to more accurately assess the transportability of the models in real-world applications, leading to the conclusion that they are “highly context-dependent,” which is a more cautious and clinically realistic interpretation, avoiding overinterpretation of the aggregated AUROC.

Our study has several limitations that should be considered when interpreting the findings. First, and most critically, is the issue of threshold heterogeneity and optimistic bias. As detailed in the “Methods” section, the performance metrics were synthesized from study-specific “optimal thresholds.” This precluded the use of threshold-independent summary measures from a bivariate model and means our pooled sensitivity and specificity are likely inflated compared to what would be achieved with a prespecified, clinically relevant cutoff. The wide PIs we report are, in part, a quantification of this inflation risk. Future primary studies should report performance at multiple, clinically justified thresholds to facilitate more meaningful meta-analysis. Second, related to the above, our statistical synthesis approach was necessitated by the data characteristics. The extreme heterogeneity and lack of threshold standardization made the preferred bivariate modeling approach unfeasible. While our use of univariate HKSJ models with PIs is a robust alternative that honestly communicates uncertainty, it does not model the correlation between sensitivity and specificity. Our subgroup and meta-regression analyses help explore sources of heterogeneity, but residual confounding is likely. Third, our search, though comprehensive, may have missed studies in other languages or in nonindexed repositories. Furthermore, we did not formally assess for publication bias using funnel plots or statistical tests, as these methods are less established and interpretable for diagnostic accuracy data with high heterogeneity. Therefore, our results may be influenced by the preferential publication of studies with positive or high-performance results.

### Clinical Significance

The methodological choices in this meta-analysis directly inform its central message. The decision to extract data at study-specific “optimal thresholds” inherently captures the optimistic bias prevalent in ML model development. The strikingly wide 95% PI for sensitivity (0.32-0.96), calculated from these potentially inflated estimates, therefore represents a conservative and realistic warning. The true performance in a new setting, after necessary recalibration to a local threshold, could fall to clinically unacceptable levels. This finding powerfully reinforces the principle that external validation is not a mere formality but a fundamental requirement to bridge the gap between algorithmic promise and clinical utility.

Clinical implementation of these models requires a shift from “universal application” to “local adaptation.” Given the wide PIs, hospitals should not adopt published models directly. Instead, we recommend a workflow of local validation and recalibration. Future research should prioritize multicenter external validation over developing new models. Where data sharing is restricted, federated learning offers a promising pathway to train robust models across diverse populations without compromising privacy.

### Conclusions

In summary, ML models demonstrate promising potential for predicting preeclampsia, rather than serving as ready-made universal solutions. While pooled analyses indicate high discriminative performance, the substantial heterogeneity (*I*²>99%) and wide 95% PIs (sensitivity 0.32-0.96) reveal significant instability in model performance across different clinical contexts. This “context dependency” was further corroborated in external validation analyses. When applied to independent populations, the model not only exhibited decreased aggregate sensitivity but also the lower bound of its PI dropped to 0.25, quantifying the substantial transplantation risk encountered in cross-center applications. Current evidence therefore supports considering ML as a potential screening adjunct, but does not yet justify its use as a universal clinical diagnostic tool. Future research should shift focus from solely pursuing new models with high AUC values to conducting rigorous multicenter external validation and recalibration of existing models, in order to establish their applicable boundaries within real-world clinical pathways.

## Appendix

Multimedia Appendix 1 PRISMA checklist.

Multimedia Appendix 2 Search strategies, baseline characteristics of included studies, and additional forest plots for subgroup analyses.

Multimedia Appendix 3 Checklists from the Prediction Model Risk of Bias Assessment Tool (PROBAST).

Multimedia Appendix 4 Workflow of the meta-analysis on machine learning models for preeclampsia prediction.

## Abbreviations

AI: artificial intelligence
AUC: area under the curve
AUROC: area under the receiver operating characteristic curve
CNKI: China National Knowledge Infrastructure
DOR: diagnostic odds ratio
HKSJ: Hartung-Knapp-Sidik-Jonkman
LR: logistic regression
ML: machine learning
NLR: negative likelihood ratio
NN: neural network
PI: prediction interval
PICO: Population, Intervention, Comparison, and Outcome
PLR: positive likelihood ratio
PRISMA: Preferred Reporting Items for Systematic Reviews and Meta-Analyses
PROBAST: Prediction Risk of Bias Assessment Tool
RF: random forest
SVM: support vector machine
TRIPOD: Transparent Reporting of a Multivariable Prediction Model for Individual Prognosis or Diagnosis
XGBoost: extreme gradient boosting
