# Supplementary material for: Machine Learning Prediction Models for Preeclampsia: Systematic Review and Meta-Analysis
Source: J Med Internet Res. 2026 Jan 19;28:e78714. doi: 10.2196/78714 (PMC12865342; doi:10.2196/78714)
Supplement: Multimedia Appendix 2 [file jmir_v28i1e78714_app2.docx]

**Multimedia Appendix 1**

##### **Table S1: Selection criteria of predictive modelling studies in PICOTS format**

|  | **Participants (P)** | **Intervention**  **(I)** | **Control (C)** | **Outcomes**  **(O)** | **Timeframe**  **(T)** | **Setting**  **(S)** | **Other limits** |
| --- | --- | --- | --- | --- | --- | --- | --- |
| **Inclusion criteria** | Pregnant women>18 years old； | ML predictive modelling: supervised, unsupervised, semi-supervised ML or combinations | Golden standards | **Primary:** metrics of discrimination ability, calibration, and classification accuracy in PE prediction  **Secondary:** important variables, intended use of models | Establish a database-2025 | **Clinical care settings** e.g. hospitals, institutions, case-control study, population-based cohort. | Language = English, Chinese |
| **Exclusion criteria** | Only study preeclampsia patients with specific severe complications (such as HELLP syndrome)； | Predictive modelling without an explicit ML approach |  | No studies have provided sufficient performance metrics for the predictive models (such as accuracy, sensitivity, specificity, etc.) |  |  | Other language |

##### **Table S2: Baseline characteristics of the 25 studies selected for the meta-analysis (data sources and participants)**

| Study ID | Country in which data was gathered | Setting | Data source | Study design | Prediction temporality | Primary outcome definition | Included PE cases |
| --- | --- | --- | --- | --- | --- | --- | --- |
| Melinte-Popescu 2023 | Romania | Hospital-based | EHRs, clinical measurements, laboratory tests | case-control study | Prognostic | ISSHP | PE/non-PE (116/116) |
| Araújo 2024 | Brazil | Hospital-based | EHRs, medical records | Case-control study | Diagnostic | ACOG | PE/Healthy (65/67) |
| Garrido-Giménez 2023 | Spain | Hospital-based | EHRs, clinical measurements, laboratory tests (sFlt-1, PlGF, NT-proBNP, uric acid) | Multicentric cohort study | Prognostic | ISSHP | PE/Healthy (207/390) |
| Torres 2024 | Mexico | Hospital-based | EHRs, questionnaires, serum biomarkers, ultrasound findings | Prospective cohort | Prognostic | ACOG | PE/Healthy (24/2926) |
| Li 2024 | China | Hospital-based | EHRs, serum samples (MAP, UtA-PI, PAPP-A, PLGF) | Prospective cohort | Prognostic | ACOG/ISSHP | PE/Healthy (210/4434) |
| Ansbacher 2022 | UK | Hospital-based | EHRs, maternal characteristics, medical history, serum biomarkers (UtA-PI, MAP, PIGF, PAPP-A) | Prospective cohort | Prognostic | ACOG | PE/non-PE  (1722/59067) |
| Zhou 2024 | China | Hospital-based | Retinal fundus images, deep learning algorithms | Prospective cohort study | Prognostic | ACOG | PE/non-PE (66/1072) |
| Tiruneh 2024 | Australia | public health networks | Routinely collected health data | Prospective cohort study | Prognostic | Clinical | PE/Healthy (1684/46566) |
| Yu 2024 | China | Hospital-based | NIPT data, clinical risk factors | Retrospective case-control study | Prognostic | ACOG | PE/non-PE (143EOPE+580LOPE/2004) |
| Roque2024 | Peru | Hospital-based | Obstetrics records and laboratory test results | Retrospective cohort | Prognostic | ACOG | PE/non-PE (35389/9235) |
| Chen2022 | Taiwan | Hospital-based | EHRs and genetic data | Retrospective cohort | Prognostic | ACOG | PE/non-PE (39/127) |
| Maric 2020 | USA | Hospital-based | EHRs, maternal characteristics, medical history, routine prenatal laboratory results | Retrospective cohort | Prognostic | ACOG | PE/non-PE (1644/14726) |
| Jhee 2019 | Korea | Hospital-based | EHRs, antenatal lab data (SBP, BUN, creatinine, platelet, etc.) | Retrospective cohort | Prognostic | ACOG | PE/Healthy (474/10532) |
| Munchel2020 | USA | Hospital-based | circulating RNA (C-RNA) in maternal blood | Retrospective cohort | Prognostic | ACOG | PE/non-PE (40/73) |
| Zhou2023 | China | Hospital-based | plasma cell-free RNA signatures and clinical data | Retrospective cohort | Prognostic | ACOG | PE/non-PE (202/715) |
| Kaya 2025 | Türkiye | Hospital-based | EHRs | Retrospective cohort | Prognostic | ACOG | PE/non-PE (50/50) |
| Xue 2023 | China | Hospital-based | EHRs, questionnaires | Retrospective cohort | Prognostic | FIGO | PE/non-PE (91/709) |
| Chen 2023 | China | Hospital-based | EHRs, questionnaires | Retrospective cohort | Prognostic | China's "Guidelines for the Diagnosis and Treatment of Hypertensive Disorders in Pregnancy (2020)" | PE/non-PE (461/864) |
| Wang2022 | China | Hospital-based | EHRs, questionnaires | Retrospective cohort | Prognostic | Diagnostic criteria from the 9th edition of Obstetrics and Gynecology (hypertension + proteinuria) | PE/non-PE (342/346) |
| Zheng 2021 | China | Hospital-based | EHRs | Retrospective cohort | Prognostic | China's "Guidelines for the Diagnosis and Treatment of Hypertensive Disorders in Pregnancy (2020)" | PE/non-PE (291/1318) |
| Kovacheva2023 | USA | Hospital-based | Electronic health record (EHR) data and linked genetic data | Retrospective cohort | Prognostic | ACOG | PE/Healthy (87/1038) |
| Sandstro¨m2019 | Sweden | Population-based cohort | EHRs, clinical records | Retrospective cohort study | Prognostic | ICD-10 | PE/non-PE (2773/59789) |
| Lv 2025 | China | Hospital-based | EHRs, clinical records | Retrospective cohort study | Prognostic | ACOG 2020 | PE/non-PE  (71E0PE，83LOPE/969) |
| Sufriyana2020 | Indonesia | chel | Nationwide health insurance dataset | Retrospective cohort study | Prognostic | NR | GDM/non-GDM |
| Wang2024 | China | Hospital-based | BPJS Kesehatan health insurance dataset | Retrospective cohort study | Prognostic | Diagnosis of preeclampsia | PE/non-PE  (1635/24074) |
| Li 2021 | China | Hospital-based | Electronic health records | Retrospective cohort study | Prognostic | ACOG | PE/non-PE (191/3568) |

##### **Textbox S1: Search terms**

| The following terms were used to search the articles in PubMed:  ("Preeclampsia"[MeSH Terms] OR preeclampsia[tiab] OR "pre-eclampsia"[tiab] OR "pregnancy-induced hypertension"[tiab] OR "hypertensive disorders of pregnancy"[tiab]) AND ( "machine learning"[tiab] OR "artificial intelligence"[tiab] OR "deep learning"[tiab] OR "neural network*"[tiab] OR "random forest*"[tiab] OR "support vector*"[tiab] OR SVM[tiab] OR "gradient boosting"[tiab] OR XGBoost[tiab] OR LightGBM[tiab] OR CatBoost[tiab] OR "logistic regression"[tiab] OR lasso[tiab] OR "elastic net"[tiab]) AND (predict*[tiab] OR model*[tiab] OR risk[tiab] OR screen*[tiab] OR diagnos*[tiab] OR prognos*[tiab]) AND (Humans[MeSH Terms]) AND (english[lang] OR chinese[lang]) AND ("2000/01/01"[Date - Publication] : "2025/02/28"[Date - Publication])  The following terms were used to search the articles in Web of Science:  TS=(preeclampsia OR "pre-eclampsia" OR "pregnancy-induced hypertension" OR "hypertensive disorders of pregnancy") AND TS=("machine learning" OR "artificial intelligence" OR "deep learning" OR "neural network*" OR "random forest*" OR XGBoost OR LightGBM OR "gradient boosting" OR "support vector*") AND TS=(predict* OR model* OR risk OR screen* OR diagnos* OR prognos*) Timespan: 2000–2025; Languages: English OR Chinese; Document Types: Article OR Proceedings Paper  The following terms were used to search the articles in IEEE Xplore:  (("preeclampsia" OR "pre-eclampsia") AND ("machine learning" OR "deep learning" OR "neural network" OR "random forest" OR "support vector" OR XGBoost OR LightGBM OR "gradient boosting") AND (predict* OR classification OR diagnosis OR risk)) Metadata filters: Year 2000–2025; Content Type: Journals, Conferences.  The following terms were used to search the articles in CNKI:  主题 = (先兆子痫 OR 子痫前期 OR 妊娠期高血压 OR 妊娠期高血压疾病) AND 主题 = (机器学习 OR 人工智能 OR 深度学习 OR 神经网络 OR 随机森林 OR 支持向量机 OR 梯度提升 OR XGBoost OR LightGBM) AND 主题 = (预测 OR 风险 OR 诊断 OR 筛查 OR 模型) 年限：2000–2025；语种：中文；文献类型：期刊/会议/学位（可按需要限定为“核心期刊”） |
| --- |

##### **Figures S1-S13: Forest plots of machine learning model for predicting PE (different subgroups).**
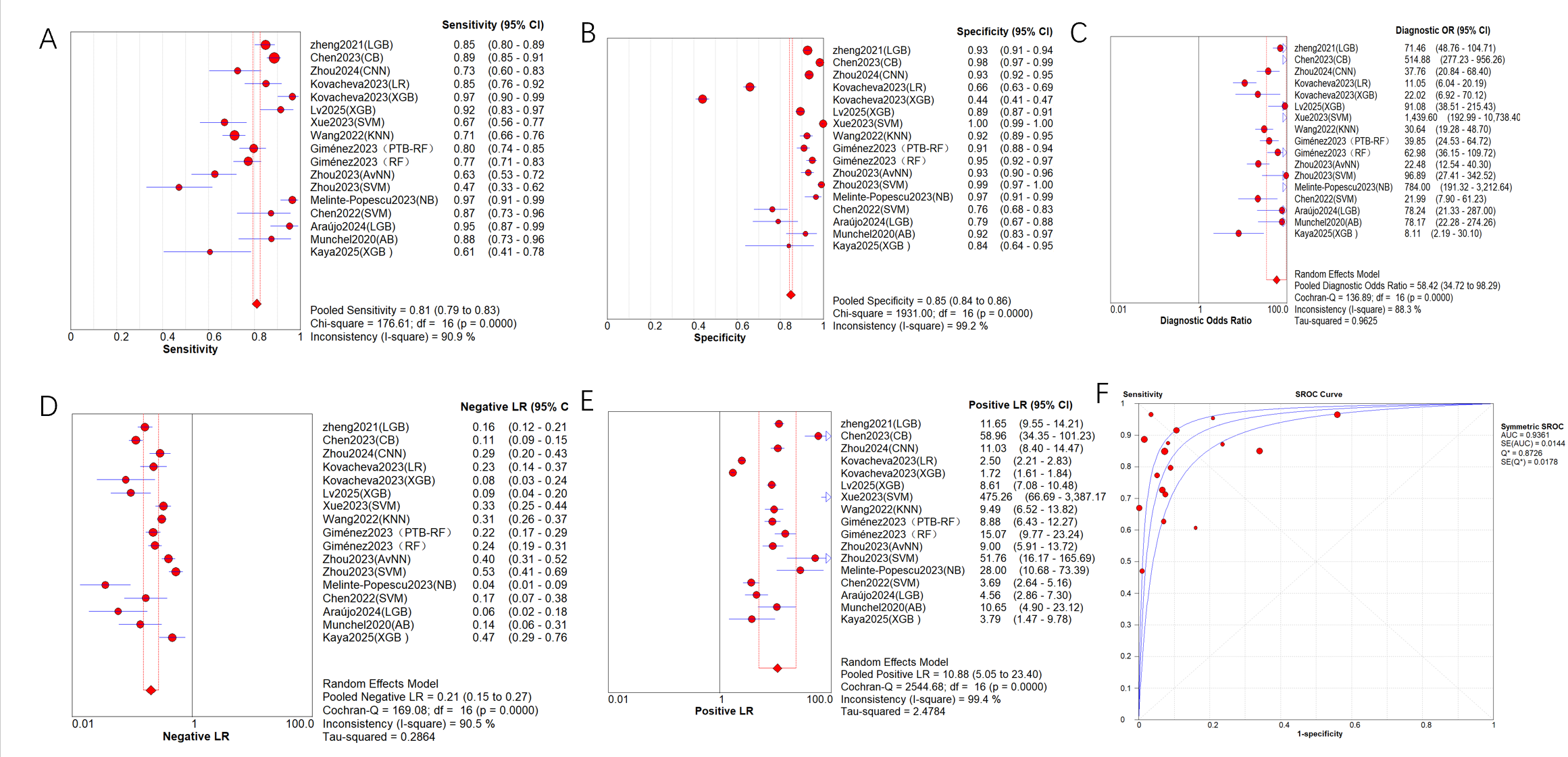


Fig.S1. A-F. predict PE with sample size＜2000


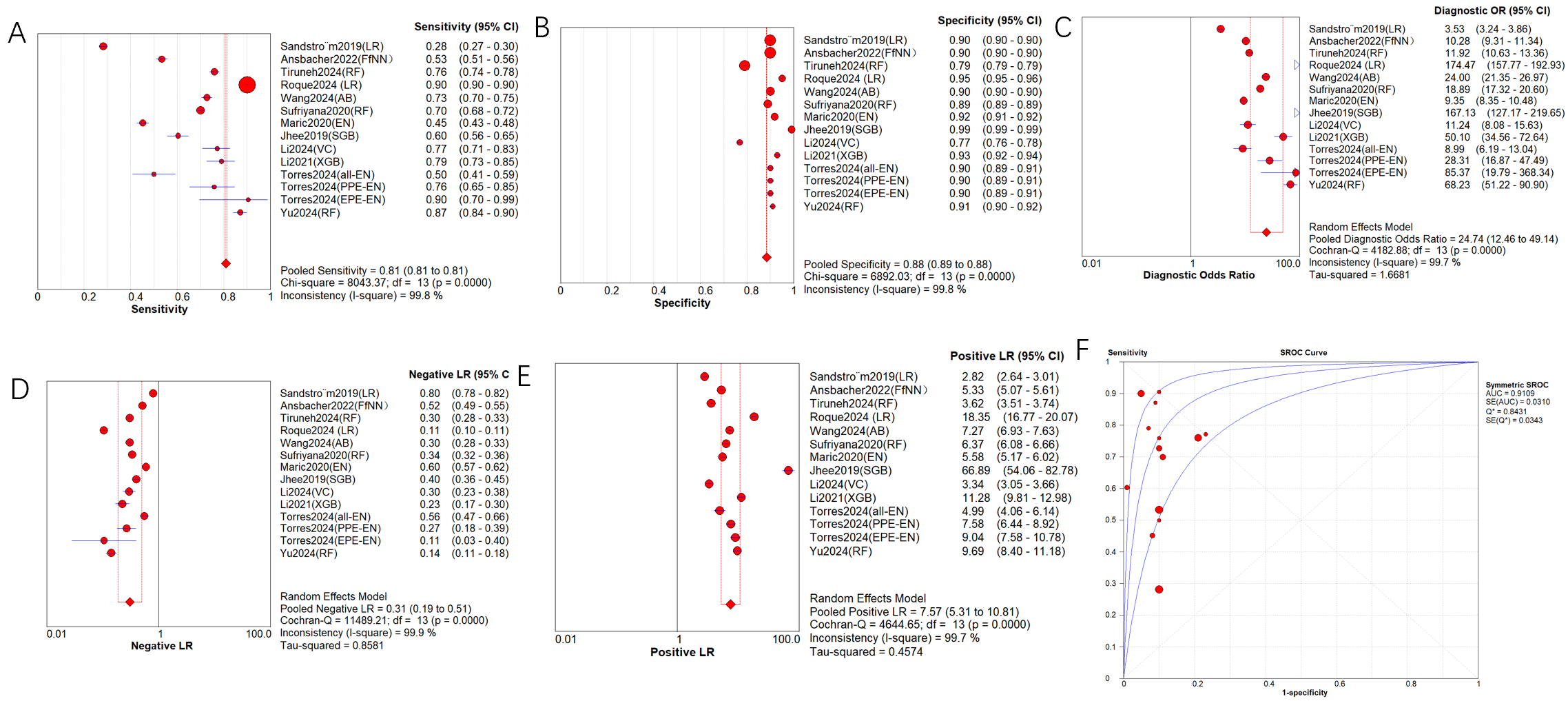


Fig.S2. A-F. predict PE with sample size≥2000


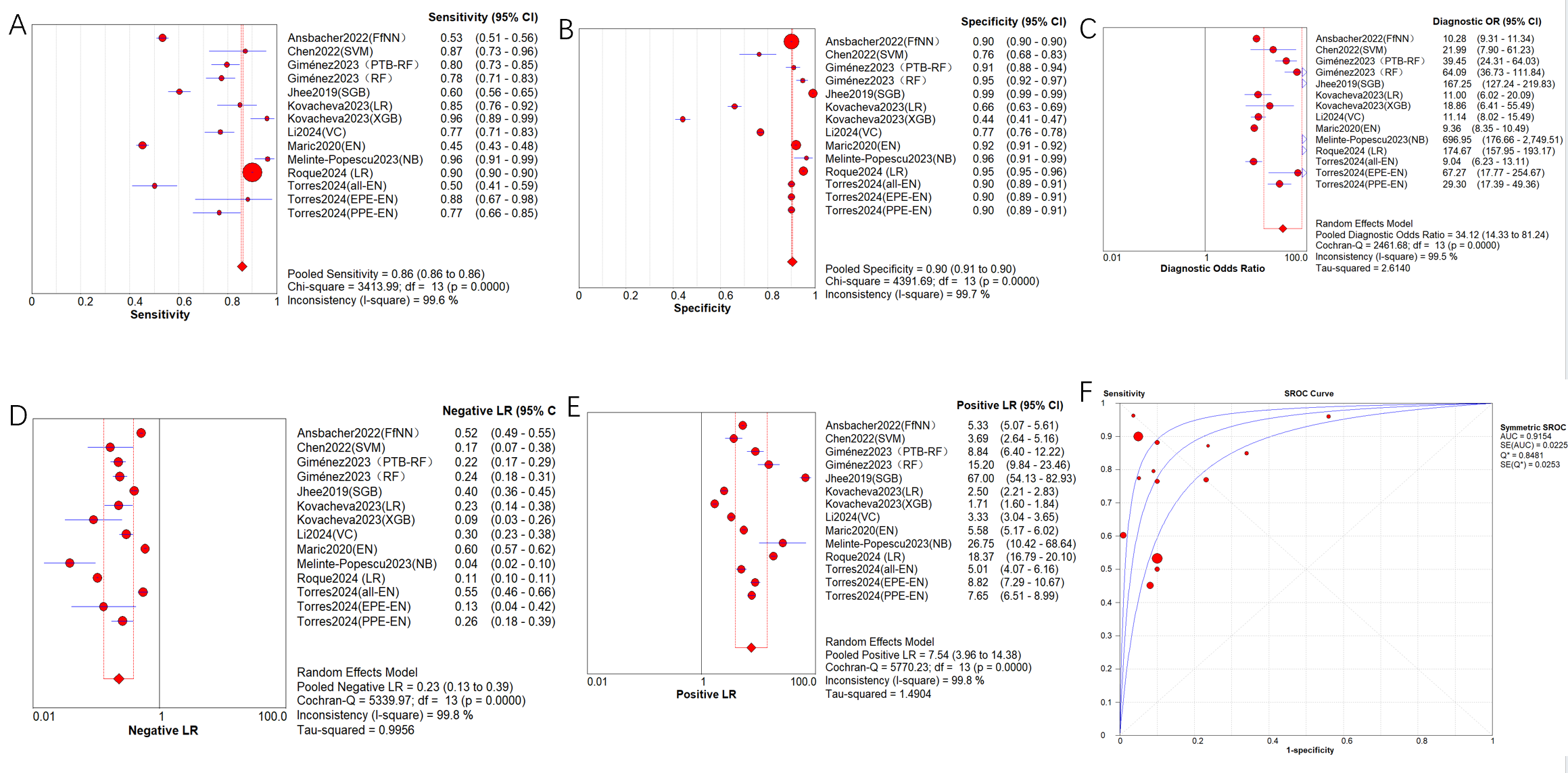


Fig.S3. A-F. predict PE with mixed of data source


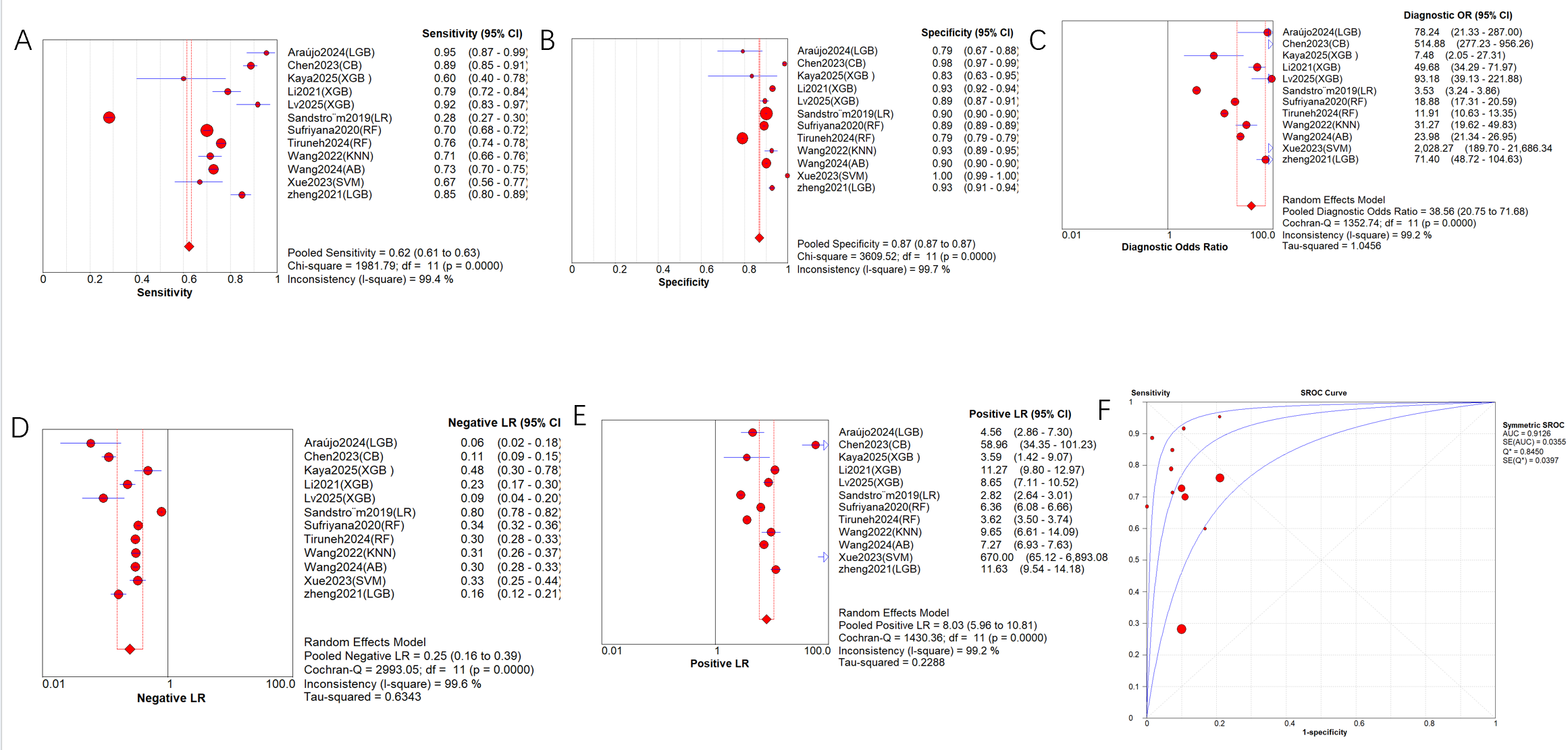


Fig.S4. A-F. predict PE with EHR of data source


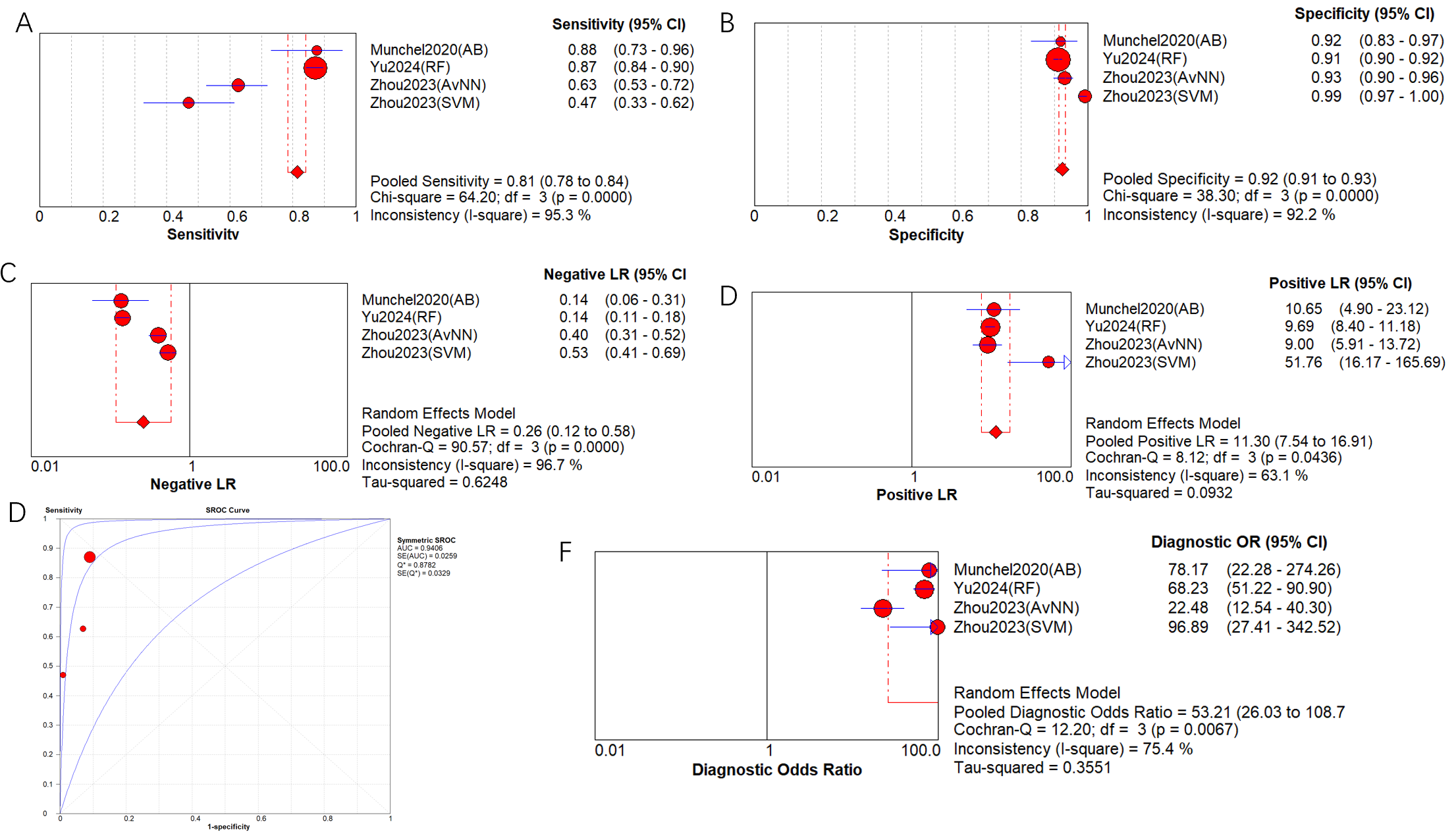


Fig.S5. A-F. predict PE with Omics of data source


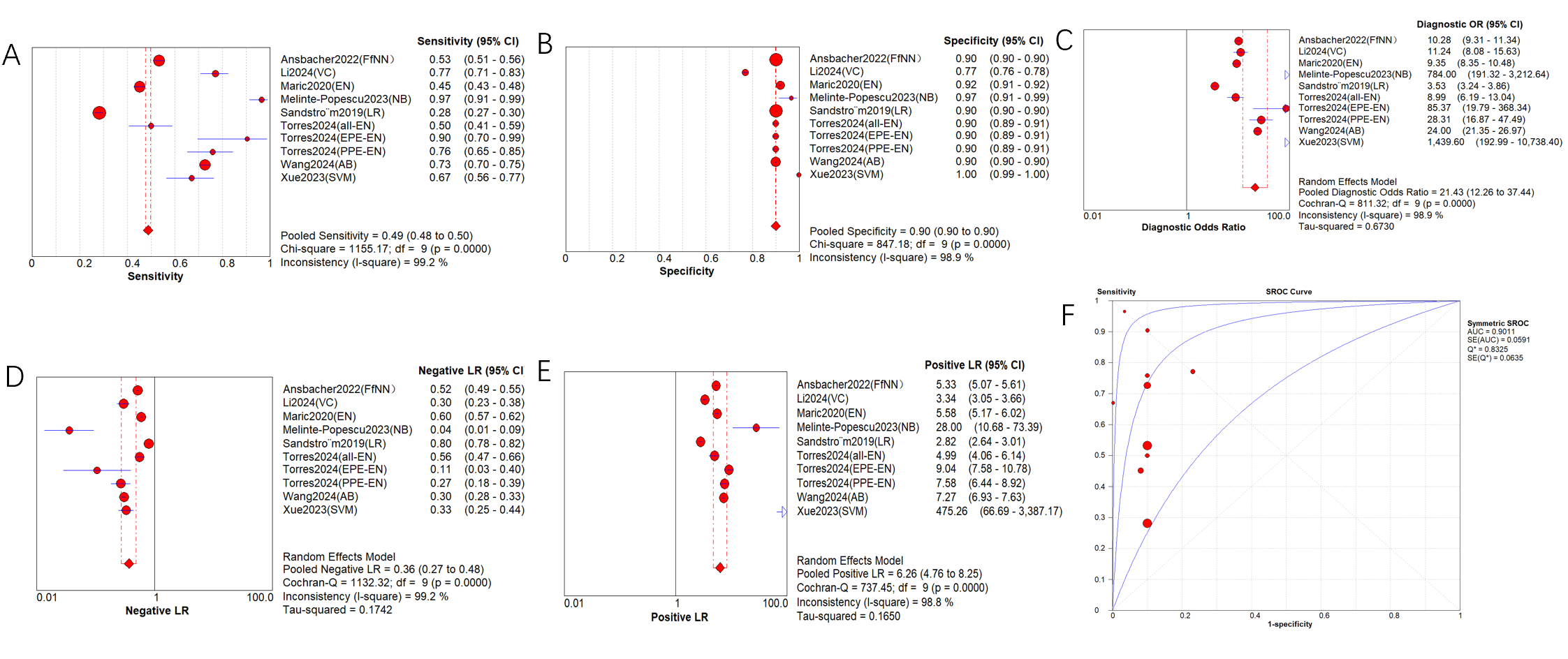


Fig.S6. A-F. predict PE with early of pregnancy window


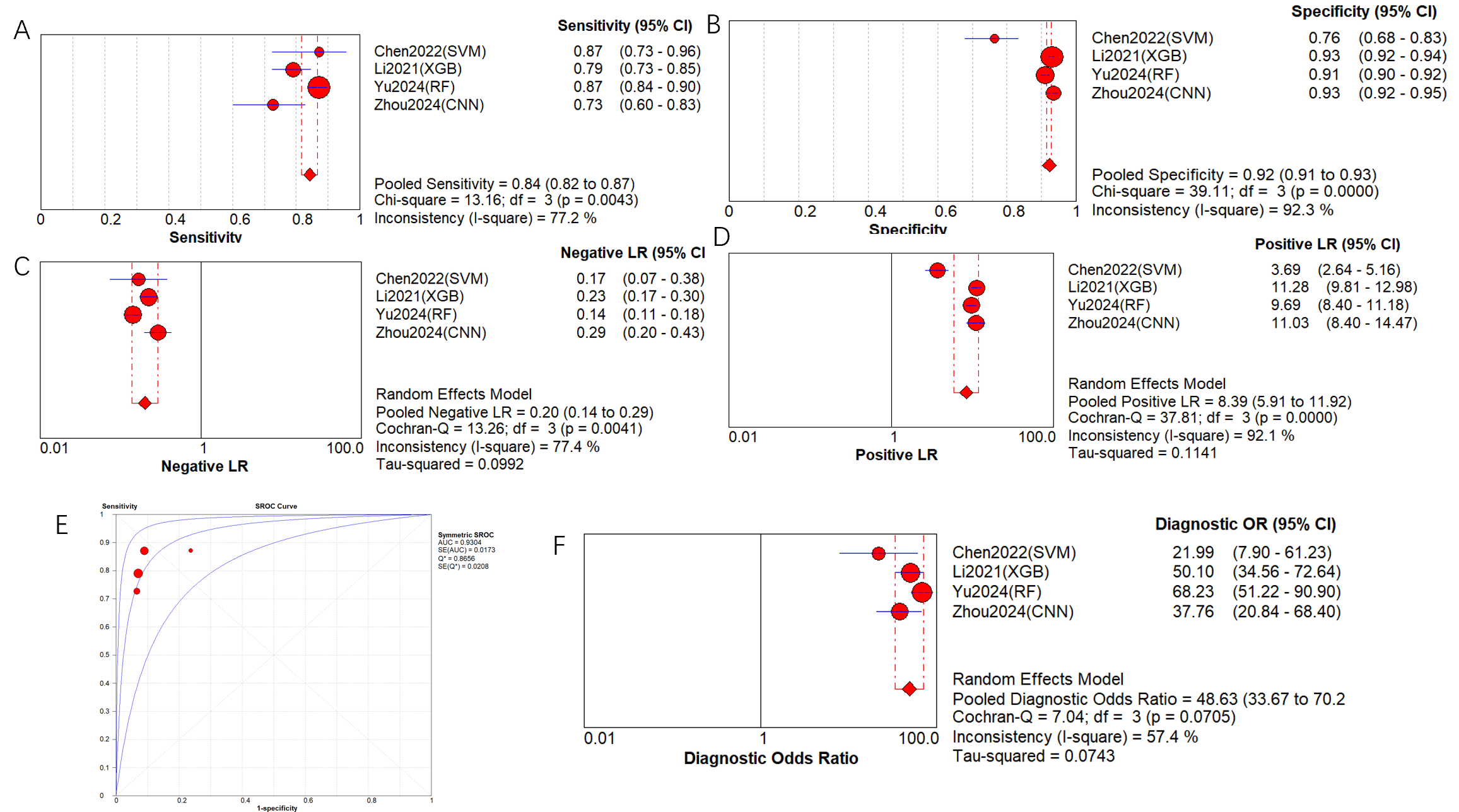


Fig.S7. A-F. predict PE with mid of pregnancy window


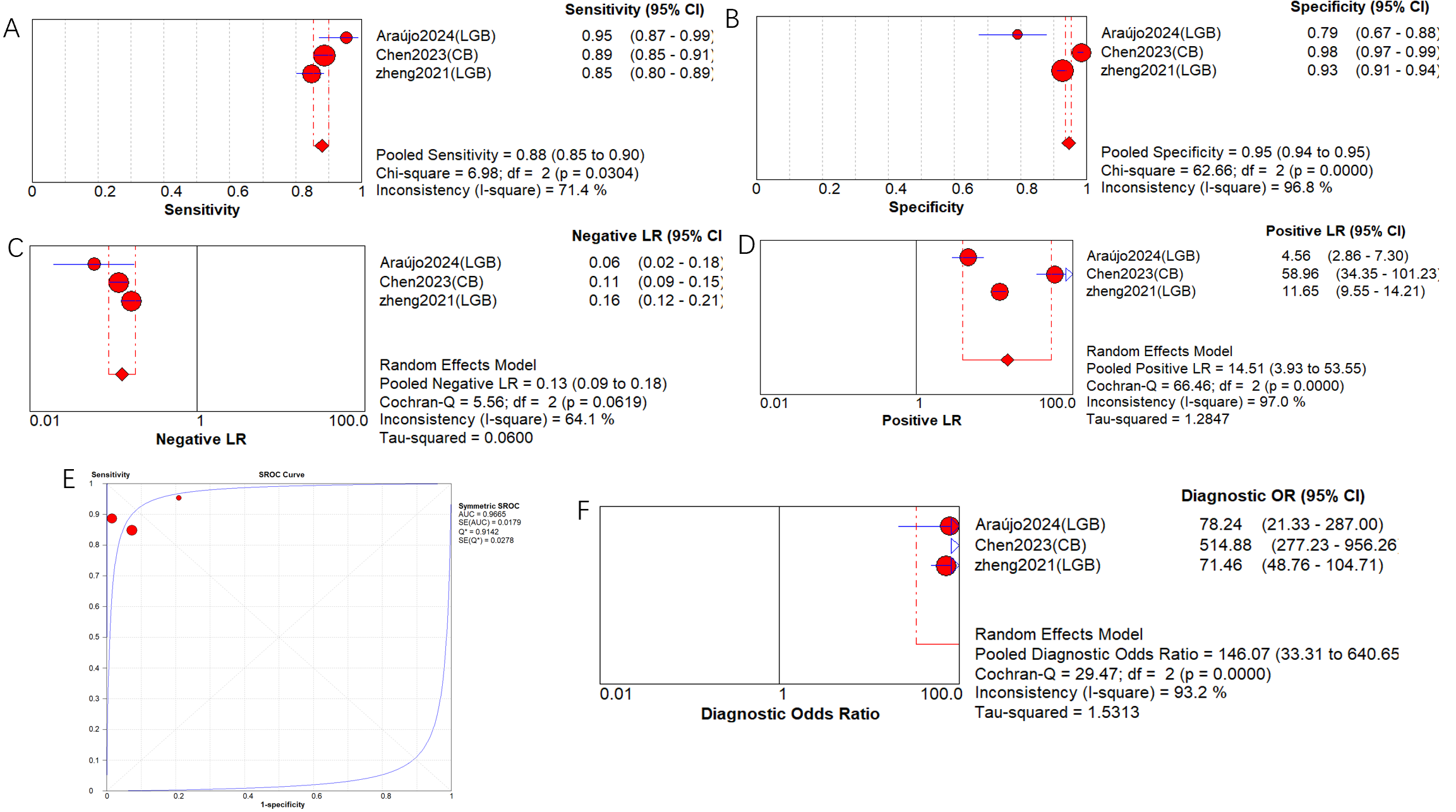


Fig.S8. A-F. predict PE with late of pregnancy window


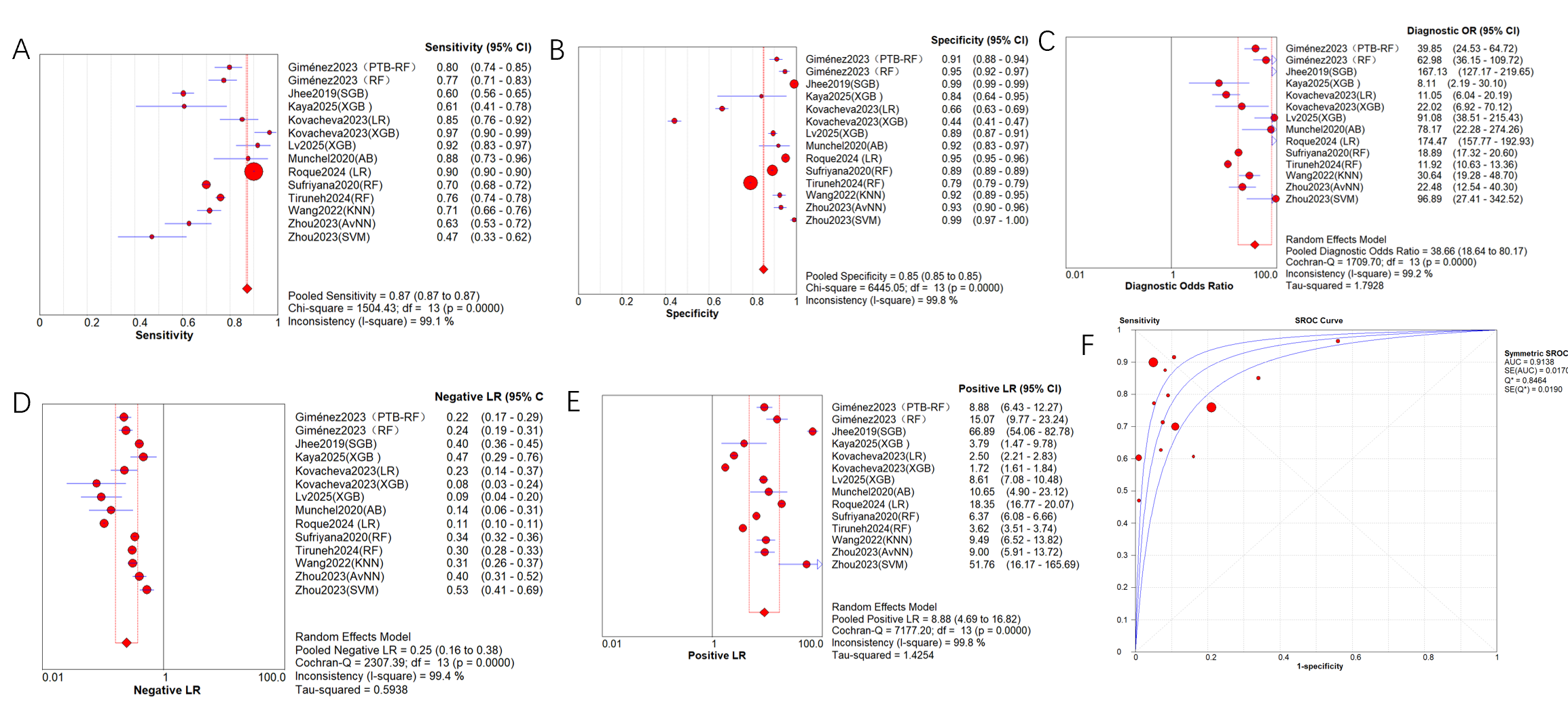


Fig.S9. A-F. predict PE with Specific of pregnancy window


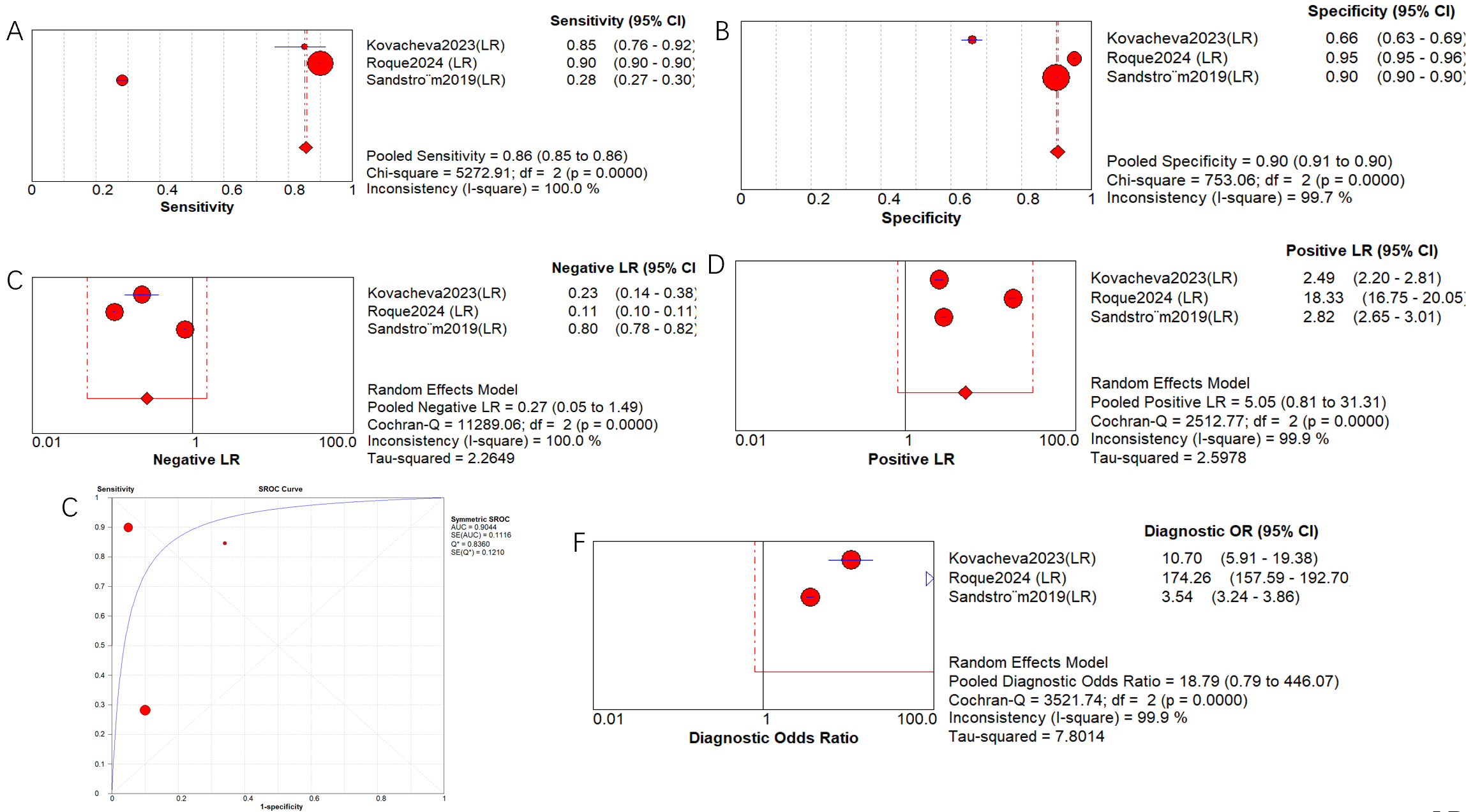


Fig.S10. A-F. predict PE with LR methods


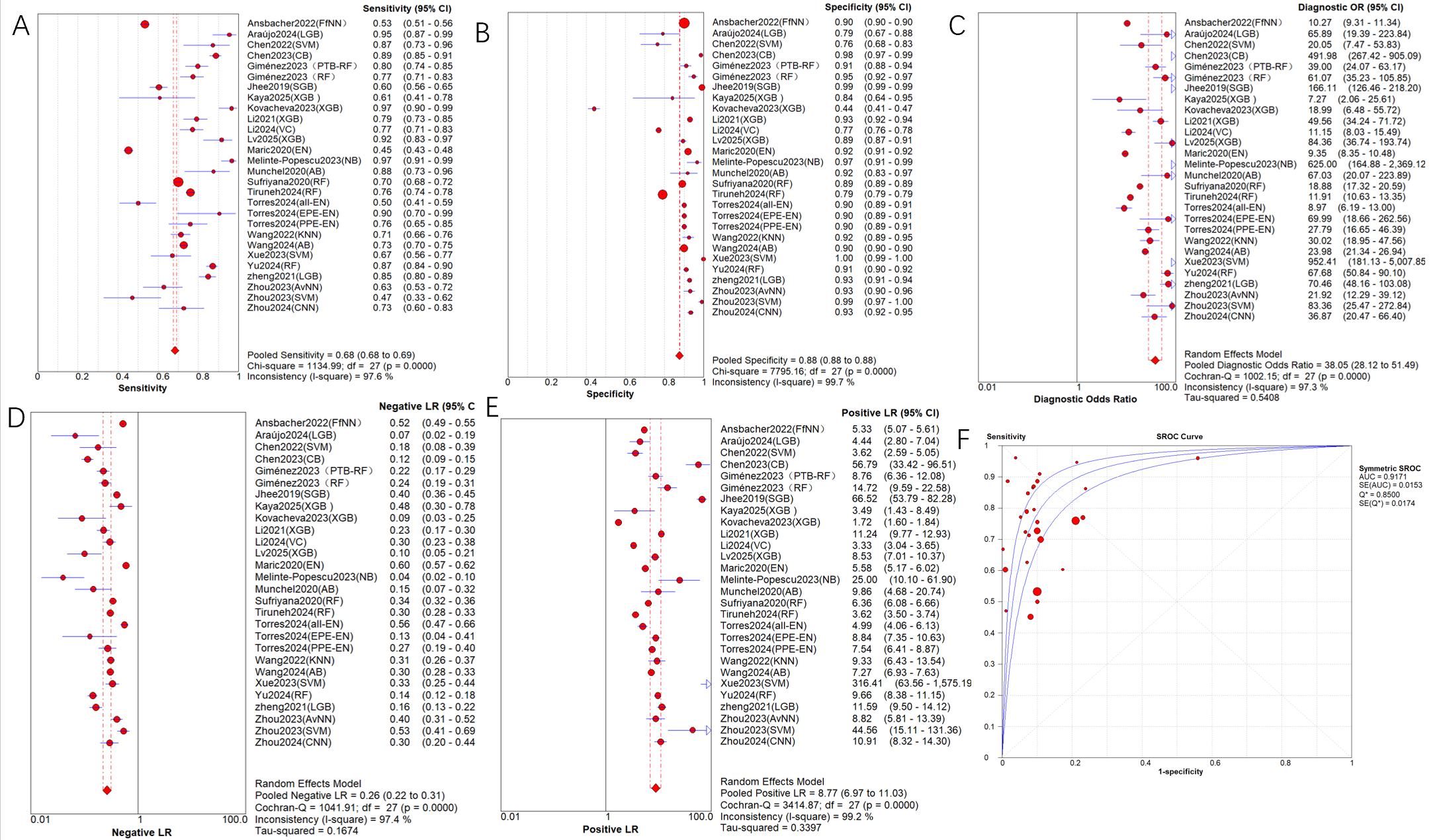


Fig.S11. A-F. predict PE with non-LR methods


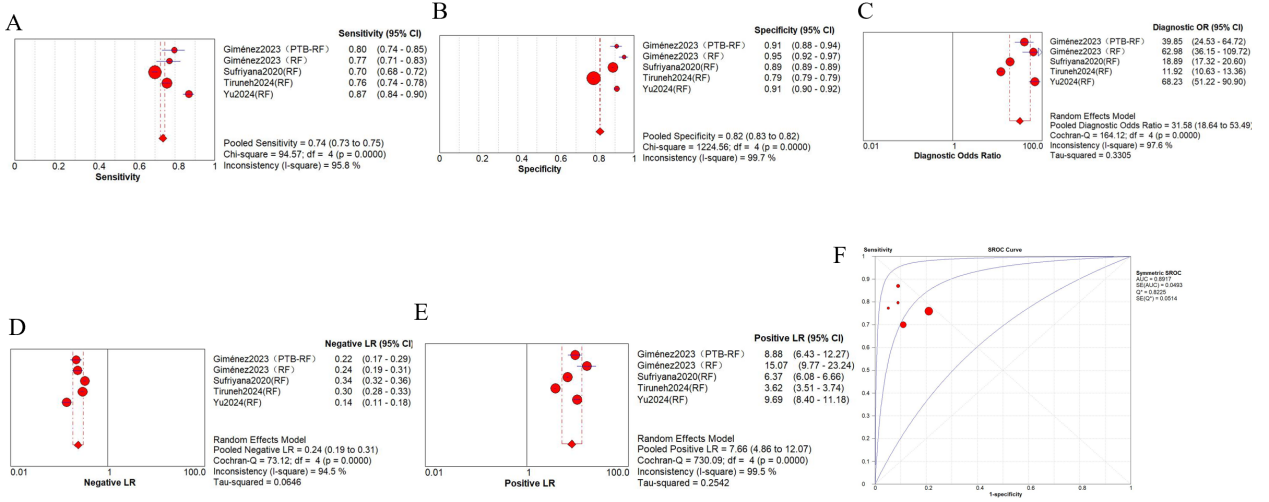


Fig.S12. A-F. predict PE with RF methods


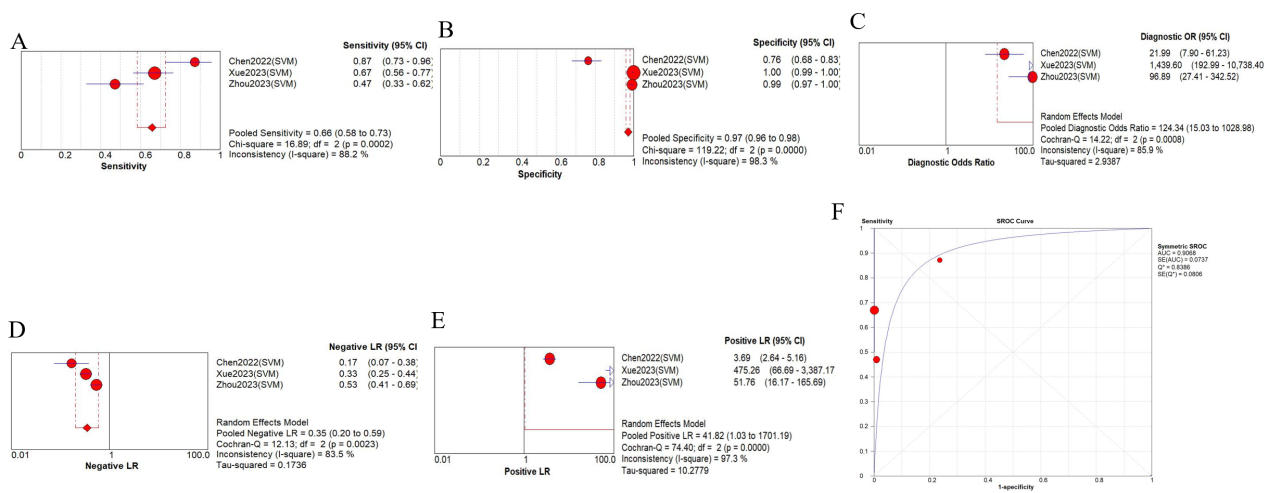


Fig.S13. A-F. predict PE with SVM methods


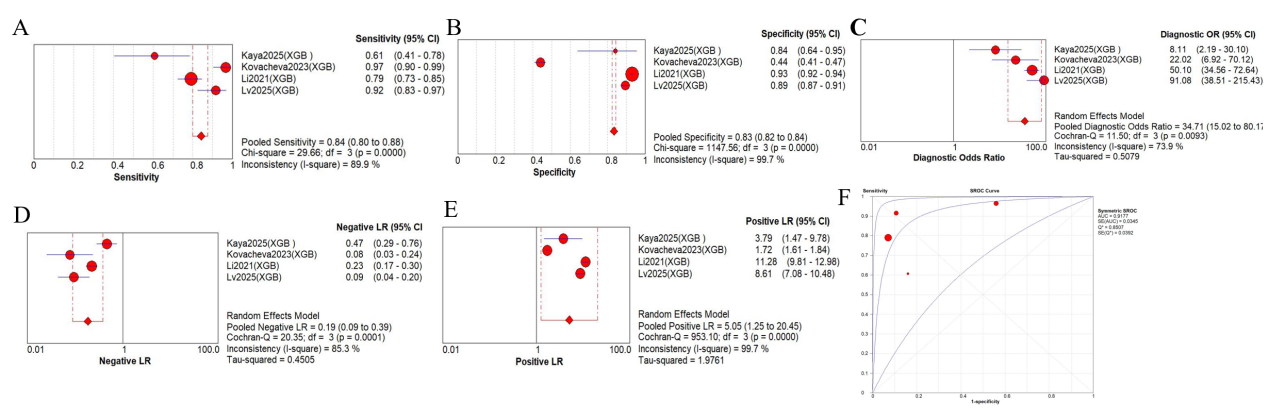


Fig.S14. A-F. predict PE with XGB methods


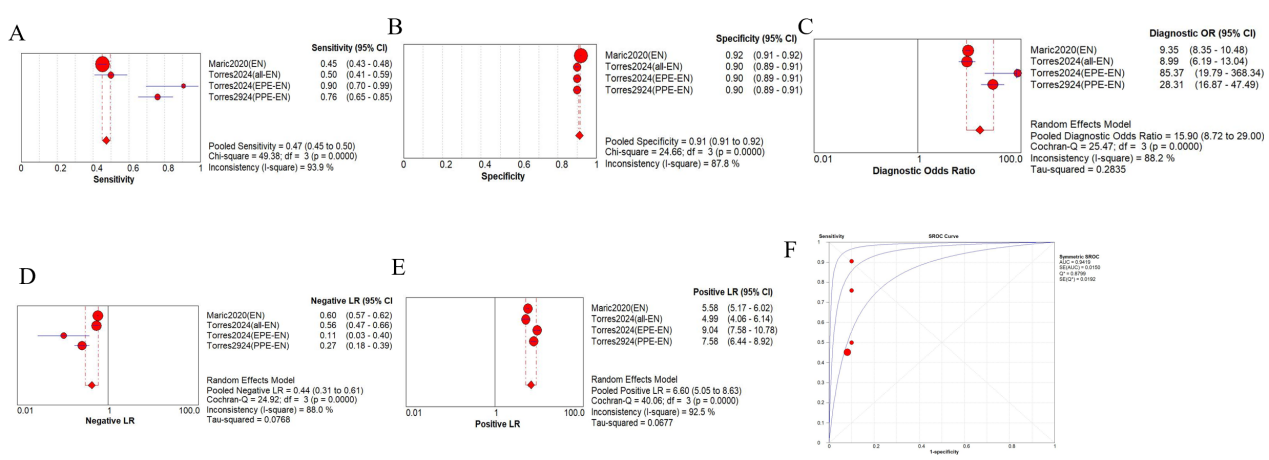


Fig.S15. A-F. predict PE with EN methods


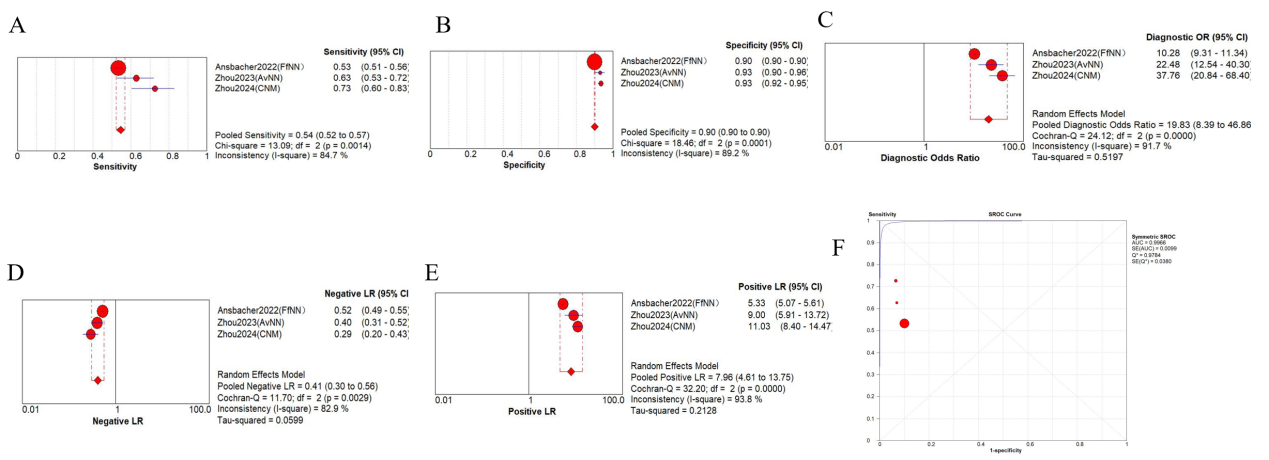


Fig.S16. A-F. predict PE with NN methods


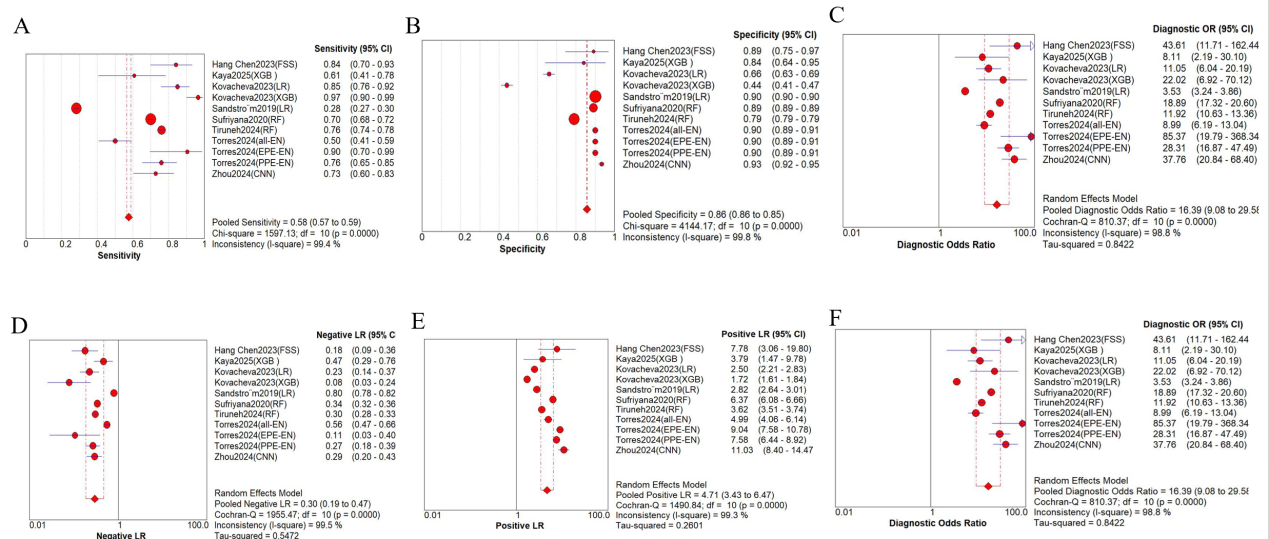


Fig.S17. A-F. predict PE with demographic information of predictor variable type


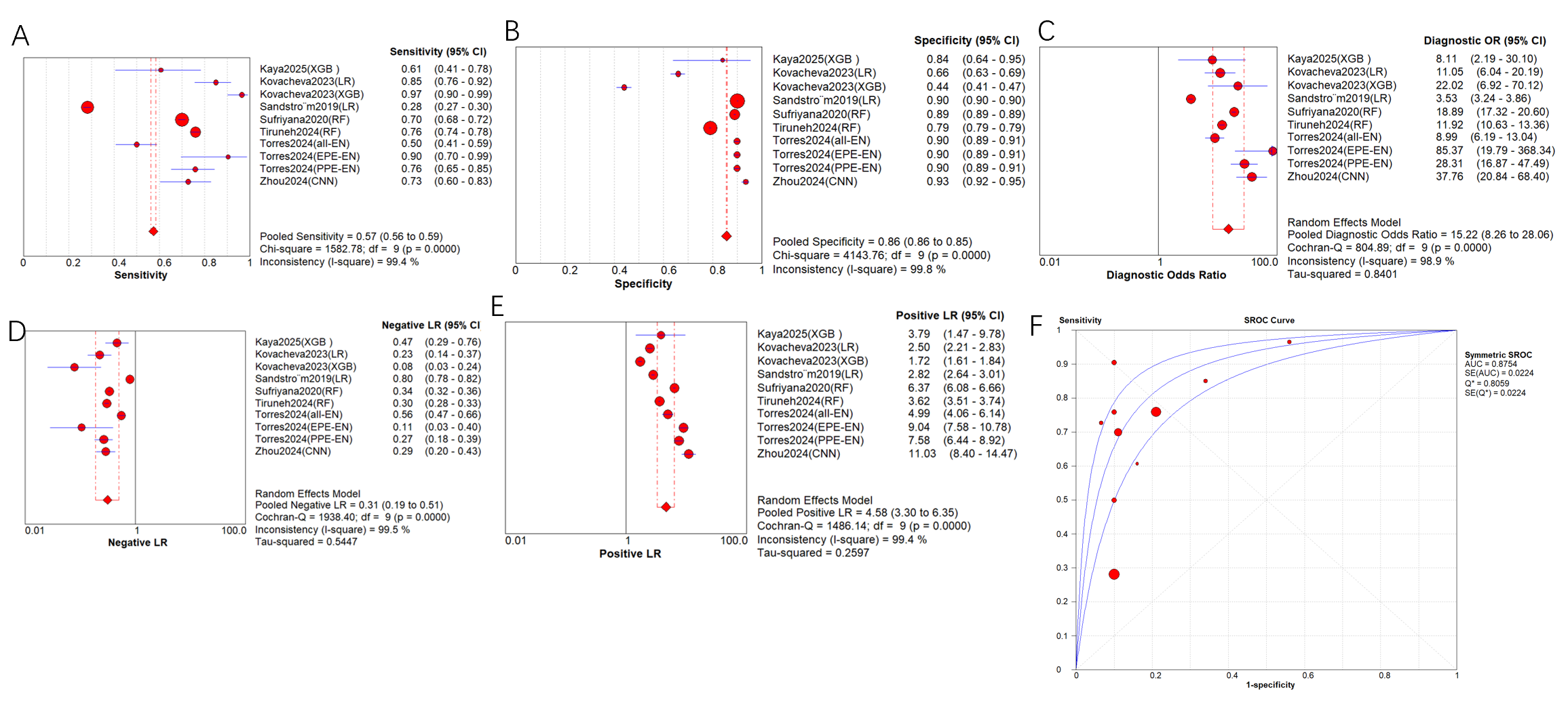


Fig.S18. A-F. predict PE with biomarker of predictor variable type


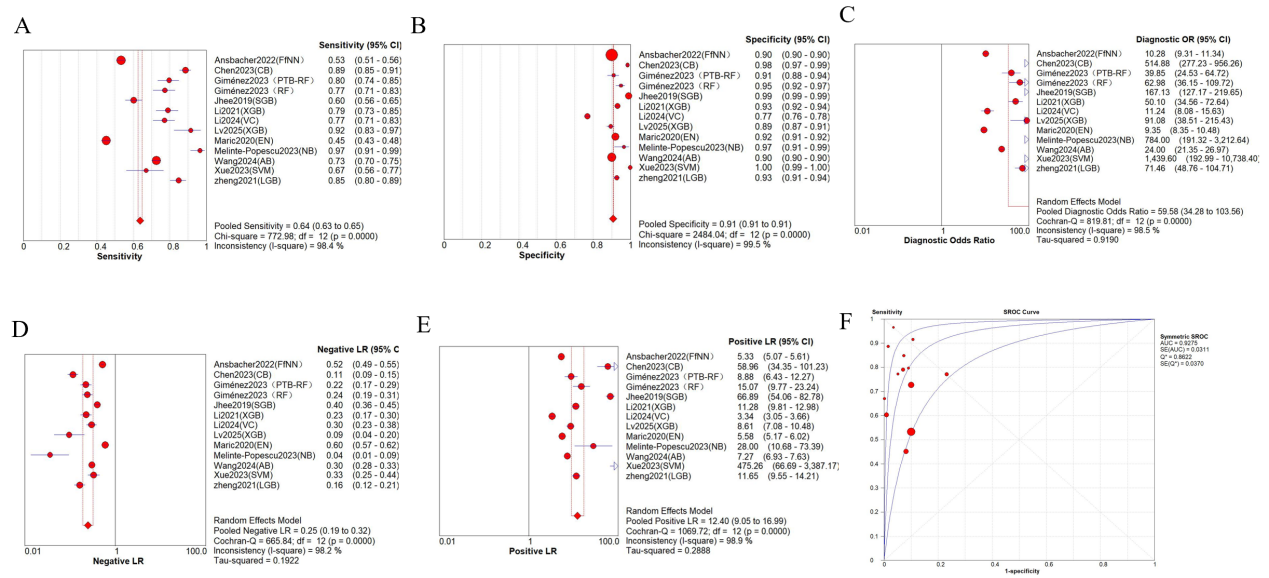


Fig.S19. A-F. predict PE with demographic information and laboratory tests of predictor variable type


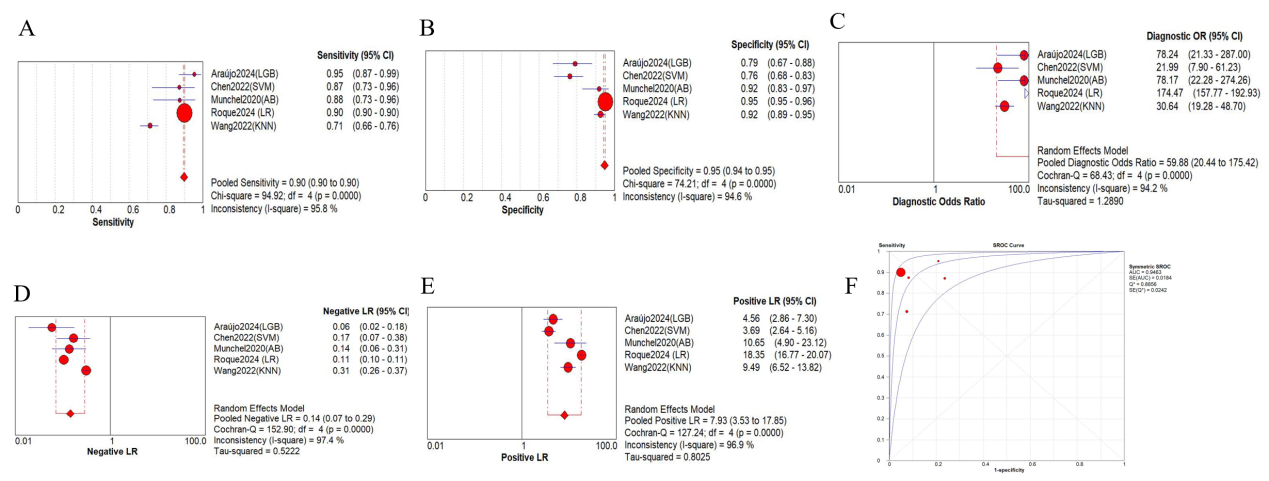


Fig.S20. A-F. predict PE with laboratory tests of predictor variable type


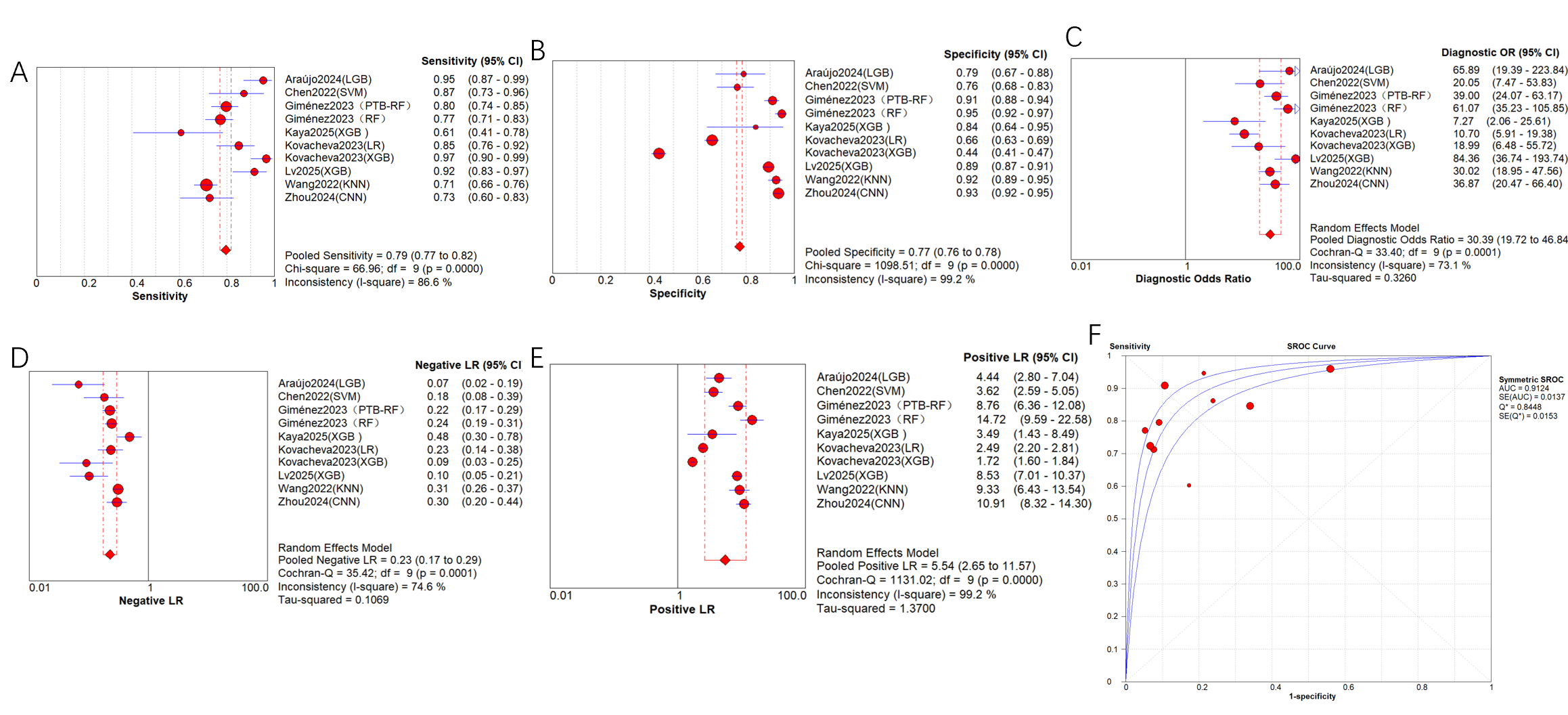


Fig.S21. A-F. predict PE with number of predictor variables＜10


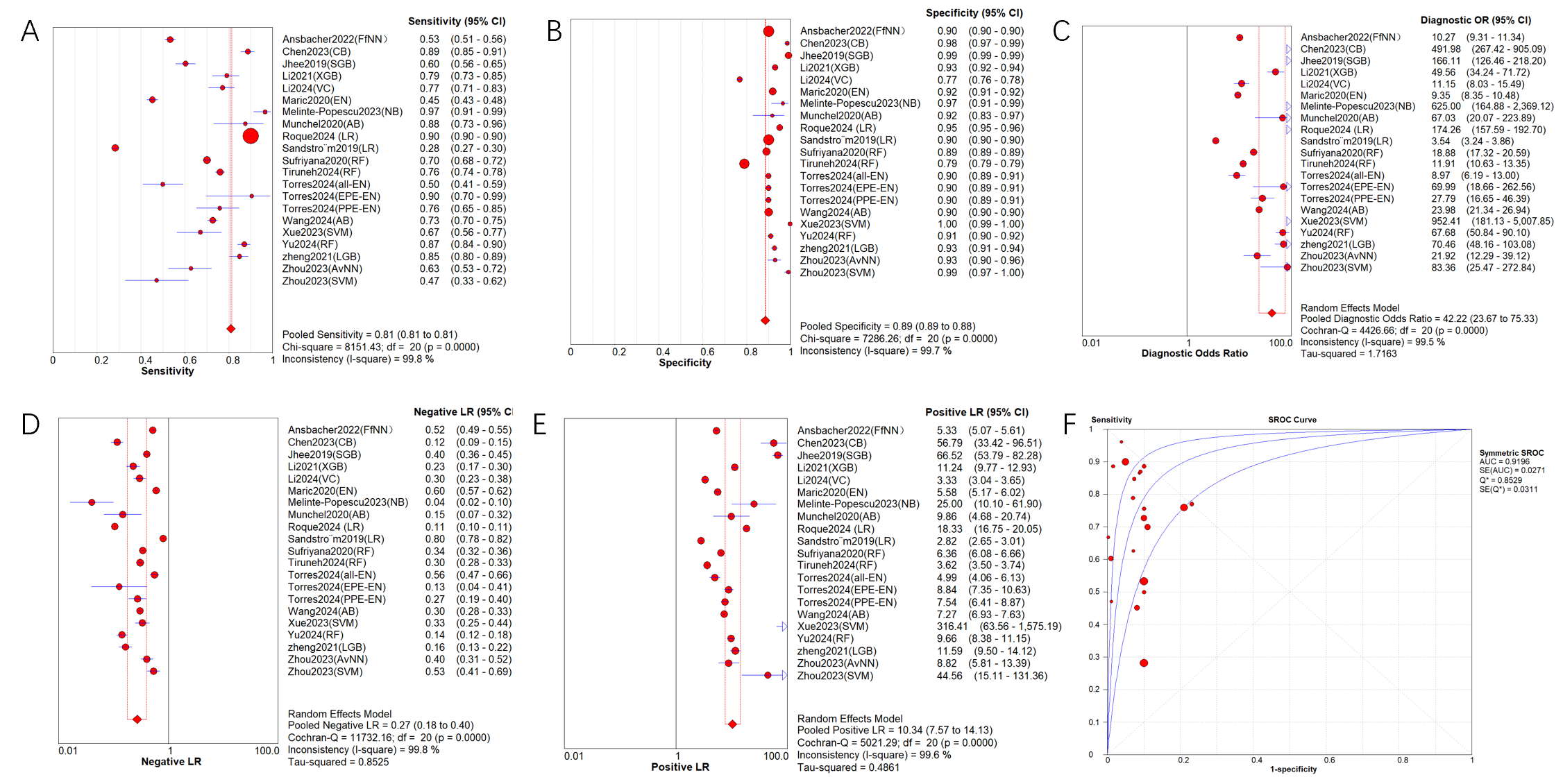


Fig.S22. A-F. predict PE with number of predictor variables≥10
