# Supplementary figures and images for: Machine Learning Prediction Models for Preeclampsia: Systematic Review and Meta-Analysis
Source: J Med Internet Res. 2026 Jan 19;28:e78714. doi: 10.2196/78714 (PMC12865342; doi:10.2196/78714)

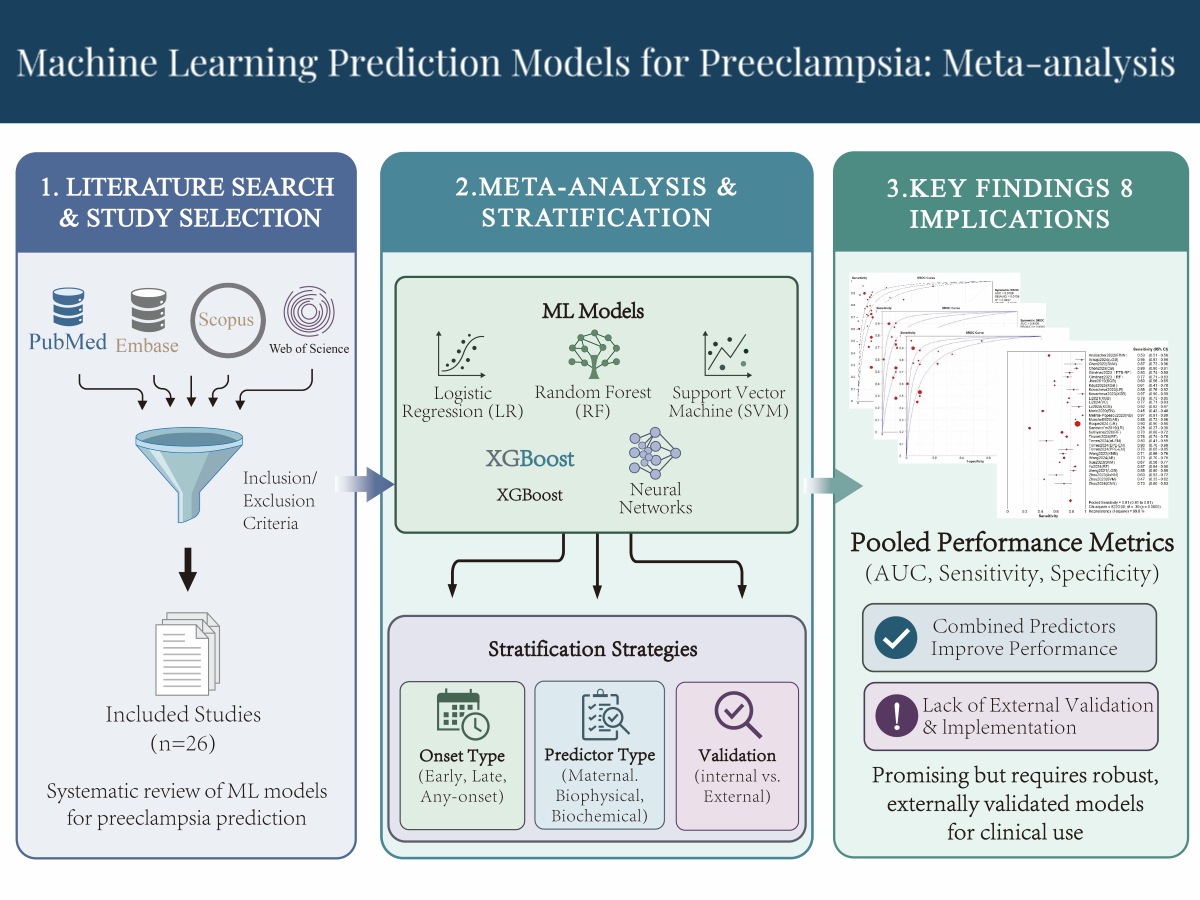

Supplement: Multimedia Appendix 4 [file jmir_v28i1e78714_app4.png]
